# Polyacrylamide Gel Calibration Phantoms for Quantification in Sodium MRI

**DOI:** 10.1002/nbm.70056

**Published:** 2025-05-07

**Authors:** Samuel Rot, Aaron Oliver‐Taylor, Rebecca R. Baker, Jennifer A. Steeden, Xavier Golay, Bhavana S. Solanky, Claudia A. M. Gandini Wheeler‐Kingshott

**Affiliations:** ^1^ NMR Research Unit, Queen Square MS Centre, Department of Neuroinflammation, UCL Queen Square Institute of Neurology, Faculty of Brain Sciences UCL London UK; ^2^ Department of Medical Physics and Biomedical Engineering UCL London UK; ^3^ Gold Standard Phantoms Limited Sheffield UK; ^4^ Centre for Cardiovascular Imaging, Institute of Cardiovascular Science UCL London UK; ^5^ Department of Brain Repair and Rehabilitation, UCL Queen Square Institute of Neurology, Faculty of Brain Sciences UCL London UK; ^6^ Quantitative Imaging Group, Department of Medical Physics and Biomedical Engineering UCL London UK; ^7^ Department of Brain & Behavioural Sciences University of Pavia Pavia Italy; ^8^ Digital Neuroscience Centre IRCCS Mondino Foundation Pavia Italy

**Keywords:** phantoms, polyacrylamide gel, relaxometry, sodium MRI, standardisation, x‐nuclei

## Abstract

Quantitative sodium (^23^Na) MRI utilises a signal calibration approach to derive maps of total sodium concentration (TSC). Agarose gel vials are often used as calibration phantoms, but as a naturally occurring substance, agarose may exhibit unfavourable qualities relating to instabilities, inconsistencies and heterogeneity. To contribute towards standardisation and methods harmonisation of quantitative ^23^Na MRI, the objective of this study was to develop and test a novel, standardisable synthetic polymer calibration phantom for in vivo quantitative ^23^Na MRI. Seven crosslinked polyacrylamide gel (PAG) samples were prepared, doped with sodium chloride (NaCl) at nominal concentrations of 10–150 mM. The sodium concentrations of all samples were estimated by volumetrics using high‐precision mass measurements. Relaxation time constants ($T_{1},T_{2}^{*}$) of all samples were measured at 3 T with a non‐localised pulse‐acquire sequence. $T_{2}^{*}$ was measured longitudinally over 14 months to assess stability. Finally, in vivo TSC quantification with PAG phantoms was demonstrated in the human brain and calf muscle on different systems, with different imaging sequences. The measured sodium concentrations of phantoms were on average 5% lower than nominal ones, owing to the unknown volumetric contribution of the solid fraction. Hence, they were reported as apparent sodium concentrations, and the apparent TSC (aTSC) was quantified in vivo. Mean relaxation time constants of ^23^Na in PAG were in the following ranges: $T_{1}$ = 27–39 ms, $T_{2s}^{*}$ = 4.8–7.1 ms, $T_{2l}^{*}$ = 16.8–18.8 ms, short fraction f = 0.64–0.77. Over 14 months, relaxation time constants were stable within 10% (above sodium concentrations of 25 mM). In vivo aTSC measurements were in the expected ranges. PAG phantoms are well suited for quantification and standardisation in ^23^Na MRI, offering tissue‐matched relaxation time constants and the intrinsic benefits of a synthetic material.

## Graphical abstract

This paper describes the preparation, testing and application of a novel type of calibration phantom for quantitative sodium MRI. The use of a synthetic, cross‐linked polyacrylamide gel substance is an important step towards standardisation in sodium MRI, overcoming inherent drawbacks of agarose‐based calibration phantoms.

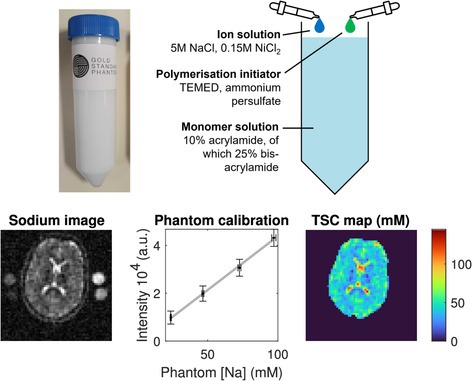

**Abbreviations**
aTSC: apparent total sodium concentration
FID: free induction decay
NUFFT: non‐uniform Fourier transform
PAG: polyacrylamide gel
ROI: region of interest
TSC: total sodium concentration
WF: water fraction
WM: white matter

## 1 Introduction

Non‐invasive in vivo magnetic resonance imaging of sodium ions (^23^Na MRI) has become an increasingly established imaging modality [1]. In vivo ^23^Na MRI provides a unique window into human (patho)physiology, as sodium ions partake in many essential processes and mechanisms (e.g., sodium–potassium exchange for osmoregulation). The sensitivity of ^23^Na MRI to numerous diseases and conditions across the body has been demonstrated [2] and continues to motivate further development of the technique. The spin‐3/2 ^23^Na nucleus is subject to quadrupolar relaxation mechanisms [3], causing fast biexponential $T_{2}$ relaxation in tissue, with a short component $T_{2s}$ = 0.5–5.5 ms and a long component $T_{2l}$ = 15–40 ms, where approximate ranges reflect diverse molecular environments across the body, among other factors [1, 2, 4]. Theory predicts an amplitude f = 0.6 of the short $T_{2s}$ fraction for sodium ions in a homogeneous, restricted molecular environment [3, 5]; this only serves as an approximation for the complex molecular environments encountered in vivo [3, 5, 6]. Usually, spin density weighted images are acquired and intensity scaled according to calibration standards of known sodium concentrations, yielding maps of the total (or tissue) sodium concentration (TSC) metric. It is a molarity concentration typically reported in units of millimolar (mM), that is, millimoles of sodium per litre of solvent (liquid volume). In tissue, TSC represents a volume fraction weighted sum of both intra‐ and extracellular sodium concentrations (10–15 mM and 140–150 mM, respectively [1, 2, 7]). The observed in vivo sodium MR signal is also modulated by the tissue water fraction (WF, approximately 0.8 averaged across human brain tissue [8, 9]), that is, the proportion of tissue containing sodium in solution.

Standards used for intensity calibration vary [4] and may be (a) internal, (b) external and acquired in the same scan or (c) external and acquired in a separate scan. For (a), calibration is performed according to intensities of internal regions maintained at a ‘known’ extracellular sodium concentration. Both CSF and the vitreous humour (VH) are commonly used in the brain. Internal standards have also been proposed for ^23^Na MRI of the kidney [10] and prostate [11]. For (b) and (c), vials typically containing agarose gel doped with different concentrations of sodium chloride are either (b) included within the target field of view or (c) acquired in a separate scan session immediately following in vivo imaging, ensuring equivalent electrical RF coil loading and hardware calibration settings.

Two important qualities of calibration standards are accuracy and stability. Accurate calibration standards require (i) relaxation time constants well matched to the tissue under investigation to reduce signal bias from residual relaxation weighting [12] and (ii) knowledge of the standard's actual sodium concentration. The signal modulations due to solid volume fractions should also be accounted for. Stability then relates to the invariance of the above features with time, batch (external standard), subject (internal standard) or environmental conditions. Meaningful relative quantification (e.g., distinction between patient and control groups) is possible with inaccurate but stable standards, reporting the *apparent* TSC, or aTSC [13]. Unstable calibration standards, however, could introduce crucial biases into large quantitative studies.

There is great heterogeneity in TSC quantification approaches across literature, as summarised in Table 1. Often favoured for convenience, internal quantification standards suffer from numerous downsides, affecting both accuracy and stability: (i) different $T_{1},T_{2}$ values compared to tissue; (ii) sodium concentration in CSF may change in pathology (e.g., migraine [31]) and even with time of day in healthy volunteers [38]; (iii) natural variations of ventricular volume, or atrophy in ageing or diseases, may affect partial volume of CSF signal across a cohort; (iv) the calibration line is poorly defined by a single non‐zero point, for which there is no consensus on the literature extracellular concentration (140 or 145 mM) or the use of a 0 mM calibration point from background noise. Laboratory measurements of the sodium concentration in CSF from spinal taps [37] or in blood serum [28] have also been used for calibration. External quantification with agarose gel is most commonly employed, as its biexponential transverse ^23^Na relaxation closely matches that in human tissue (improving accuracy), and the possibility of subject‐specific bias is reduced. Different concentrations of gelling agent (2%–5%) have been used for ^23^Na MRI of the brain, even in individual exams, altering the relaxation time constants of phantoms [39, 40, 41]. Moreover, both agar and agarose (a component of agar) are commonly utilised as gelling agent, introducing further discrepancies in quantification methods as their microstructural properties are likely to differ, for example, giving rise to different ^1^H relaxation time constants [42]. As a naturally occurring substance, agar also contains impurities; aside from heterogeneous and inconsistent properties [43], the presence of residual sodium chloride from seawater [43] is particularly concerning for meaningful TSC quantification. Accuracy may be further compromised by evaporation during gel preparation, introducing uncharacterised deviations from nominal sodium concentrations of samples [4]. Finally, degradation by formation of mould, bubbles and cracks is not uncommon in agar and agarose phantoms [4, 44], impacting their stability.

**TABLE 1:** The intensity calibration methods used for total sodium concentration (TSC) quantification in a selection of recent ^23^Na MRI publications, in chronological order. Studies were chosen to reflect the diversity of preferred calibration standards and concentrations across groups and centres but also anatomy.

Authors	Year	Method	Substance	^23^Na concentrations (mM)	Anatomy	Ref
Mellon et al.	2009	Int., CSF		140	Brain	[14]
Inglese et al.	2010	Ext., same scan	4% agar	50, 100	Brain	[15]
Haneder et al.	2011	Ext., same scan	2% agarose	103, 154 (0.6%, 0.9%)	Kidney	[10]
Hausmann et al.	2012	Ext., same scan	2% agarose (×2), aqueous NaCl (×1)	103, 154, 154 (0.6%, 0.9%, 0.9%) respectively	Prostate	[16]
Zaaraoui et al.	2012	Ext., same scan	2% agar	50, 50	Brain	[17]
Reetz et al.	2012	Ext., same scan	2% agarose	100, 150	Brain	[18]
Madelin et al.	2012	Ext., same scan	4% agar	0, 150, 200, 250, 300	Cartilage	[19]
Qian et al.	2012	Int., CSF		0, 145	Brain	[20]
Paling et al.	2013	Ext., same scan	4% agar	33, 66	Brain	[21]
Solanky et al.	2013	Ext., separate scan	Aqueous NaCl	44.8	Spinal cord	[22]
Kopp et al.	2013	Ext., same scan	Aqueous NaCl	10, 20, 30, 40	Leg muscle	[23]
Romanzetti et al.	2014	Int., CSF		0, 145	Brain	[24]
Thulborn et al.	2015	Ext., separate scan	3% agarose	30, 70, 110	Brain	[25]
Eisele et al.	2016	Ext., same scan	2% and 5% agar	50, 154	Brain	[26]
Wang et al.	2017	Ext., same scan	Aqueous NaCl	10, 20, 30, 40	Leg muscle	[27]
Adlung et al.	2018	Ext., same scan; int. VH; int. CSF0^a^	2% agarose	50, 100 (agarose); 145^b^ (internal references)	Brain	[28]
Barrett et al.	2018	Ext., separate scan	4% agar	7–160 (5 phantoms)	Prostate	[29]
Gerhalter et al.	2019	Ext., same scan	5% agarose	20, 40	Leg muscle	[30]
Meyer et al.	2019	Ext., same scan	2%, 5% agarose	50, 154	Brain	[31]
Deen et al.	2019	Ext., same scan	4% agar	20, 80	Abdomen	[32]
Gerhalter et al.	2021	Int., VH		0, 145	Brain	[33]
Zaric et al.	2021	Ext., same scan	2% agarose	77, 154	Breast	[34]
Stobbe et al.	2021	Ext., same scan	5% agar	64, 64	Brain	[35]
Mendili et al.	2022	Ext., same scan	2% agar	50, 50	Brain	[36]
Wilferth et al.	2022	Int., CSF		147.6 ± 2.2^c^	Brain	[37]

Abbreviations: CSF, cerebrospinal fluid; ext., external; int., internal; VH, vitreous humour.

^a^ Study performed a comparison of different quantification approaches.

^b^ If available, sodium concentrations from blood serum measurements were used for calibration.

^c^ Determined by laboratory analysis of spinal taps.

The measurement heterogeneity [45] and wide range of literature reported TSC values (e.g., 20–60 mM for TSC in healthy cerebral white matter [1]), as well as the lack of consensus on quantification approaches, all motivate the need for standardisation in ^23^Na MRI. Hence, the aim of our study was to develop and test a novel ^23^Na MRI quantification phantom that fulfils the following criteria for accuracy, stability and practicality: similar ^23^Na MRI relaxation properties to tissue, synthetic material, resistance to degradation and longitudinal stability, structural homogeneity and highly viscous or solid state of matter (to avoid settling time and vibration effects). We identified synthetic, cross‐linked polyacrylamide gel (PAG) as a candidate material. Therefore, this work presents (i) a method for preparing PAG phantoms with suitable ^23^Na relaxation properties, (ii) an approach for estimating the sodium concentration of samples, (iii) the phantom relaxation time constants over 14 months and (iv) their use for quantitative in vivo ^23^Na MRI.

## 2 Methods

### 2.1 Phantom Preparation

#### 2.1.1 Gel Formation

**FIGURE 1:**
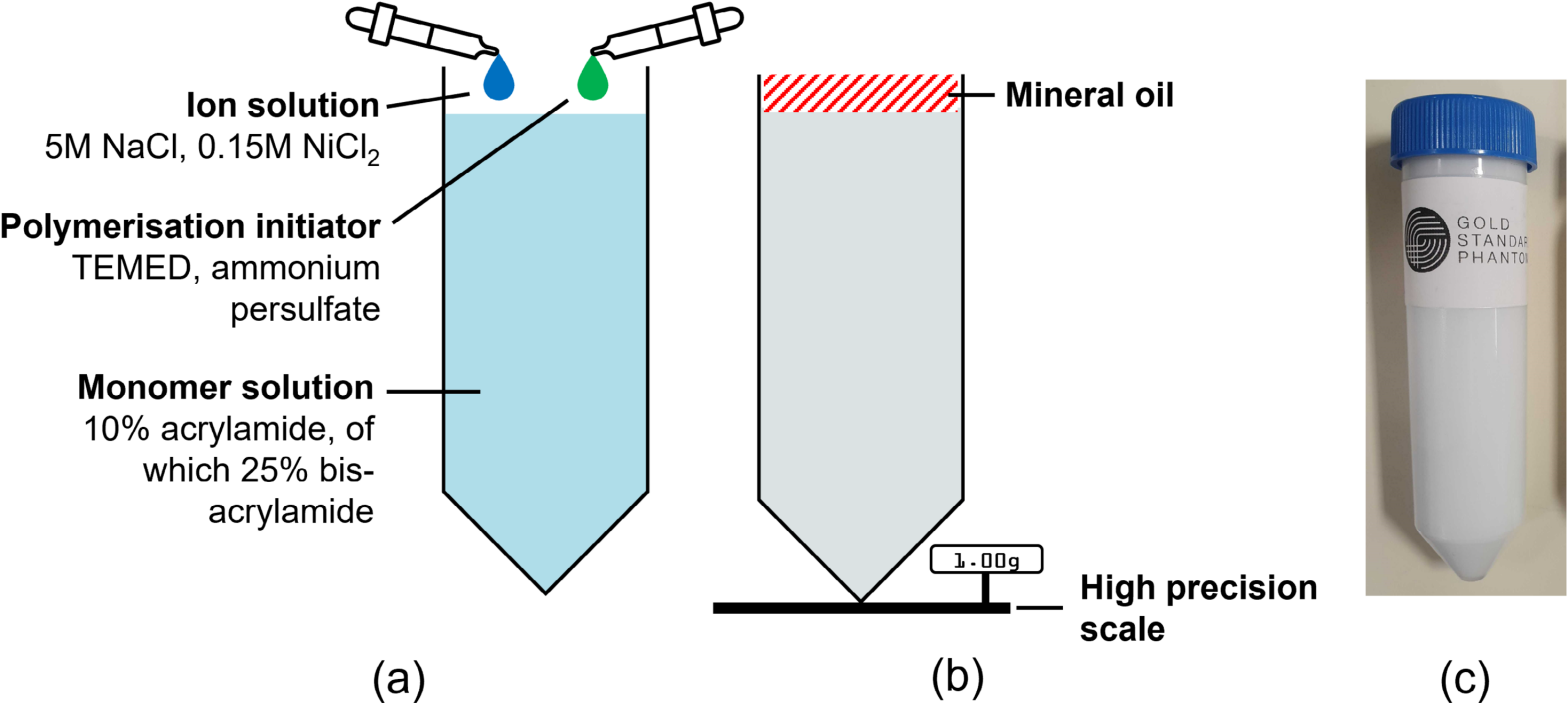
An overview of the phantom preparation procedure. Before polymerisation, (a) shows the three essential ingredients, comprising a monomer solution, to which small volumes of ion solution and polymerisation initiator are added (TEMED is tetramethylethylenediamine). After polymerisation, (b) shows estimation of the set gel volume by measuring the mass of mineral oil added to fill the vial to the brim. A photograph of the final, polymerised gel phantom is shown in (c).

**FIGURE 2:**
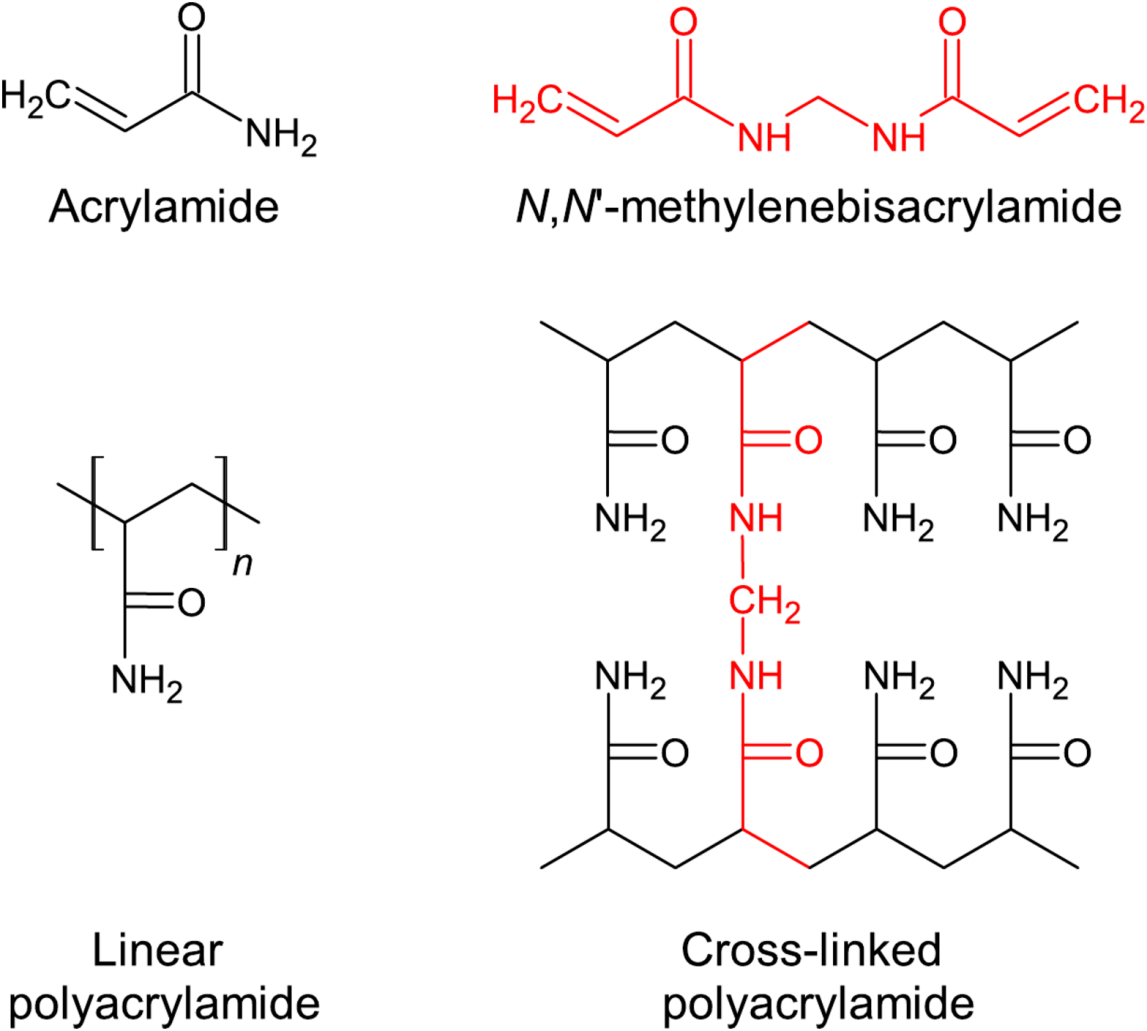
Schematic chemical diagrams of the monomer compounds acrylamide and *N,N′*‐methylenebisacrylamide (bis‐acrylamide, the cross‐linker). Unlike simple linear polyacrylamide, which forms in conventional polymer chains, cross‐linked polyacrylamide forms into so‐called polymer networks by conjoining acrylamide chains via bis‐acrylamide cross‐linkers. Their placement is irregular and random, such that cross‐linked polyacrylamide cannot be represented by a repeated polymer unit, unlike linear polyacrylamide.

Two stock solutions were prepared in batches using deoxygenated water: first, a monomer solution at a concentration of 10% acrylamide, of which 25% was bis‐acrylamide cross‐linker, and second, a highly concentrated ion stock solution at 5 M NaCl, 0.15 M NiCl_2_ (the latter for proton contrast). The monomer solution also included: 0.1% w/w ProClin 150 (Sigma) as preservative and 0.16% w/w TRIS (trisaminomethane), 1.37% w/w TRIS hydrochloride as buffers [46], achieving a physiological pH of 7.4. Then, each vial (50‐mL Falcon Conical Centrifuge Tube) was filled with monomer solution and doped with small, varying quantities of ion solution, to formulate seven samples at the following nominal sodium concentrations: 10, 25, 50, 75, 100, 125 and 150 mM. Utilising small amounts of highly concentrated ion solution maintains an approximately constant monomer concentration across all samples. Finally, 0.25 g of ammonium persulfate and 57 μL of TEMED (tetramethylethylenediamine) were added to each vial, acting as a free radical system to initiate polymerisation [46]. All preparation was carried out at room temperature using mass measurements with a high‐precision scale (Adam Equipment Nimbus NBL 4602i, ±0.01 g). Unless stated otherwise, reagents and chemicals were sourced from Glentham Life Sciences. The preparation steps are summarised schematically in Figure 1. Further, Figure 2 shows the chemical structures of monomer compounds, simple and cross‐linked PAG, illustrating the role of the cross‐linker additive to form a polymer network.

In addition, a 3% agarose sample at a nominal sodium concentration of 85 mM was prepared. This was to provide a reference for relaxation measurements, as biexponential transverse relaxation of ^23^Na in agarose gels is well established [5, 47]. Agarose and sodium concentrations were chosen to give the desired relaxation characteristics [1] and an intermediate value within the range of PAG phantom concentrations, respectively.

##### 2.1.1.1 Concentration Measurement

The gels were prepared to nominal sodium concentrations using mass measurements, allowing the precise evaluation of the mass of sodium present in each vial, in grams. However, as the samples were prepared by dilution of different quantities of the 5 M NaCl stock solution with the monomer solution of unknown density, without performing volumetric measurements, the actual sodium concentration (molarity) of samples was unknown. Therefore, after sample preparation, the final gel volume was measured and utilised to calculate the sodium concentration of the samples. Because the volumetric contribution of the solid fraction is not known, the measurements must be considered *apparent* values. Ignoring relaxation effects, a PAG sample of a certain *apparent* sodium concentration will produce the same intensity MRI signal as a saline sample of the same sodium concentration.

To quantify the total gel volume, the empty portion of the vial was filled with inert mineral oil to the brim and its mass was measured with a high‐precision scale (Ohaus PX5202, ±0.01 g). After experimentally determining the oil density, it was possible to derive an estimate, as well as uncertainty, for the gel volume and the apparent sodium concentration. The uncertainty in assessing ‘full to the brim’ was characterised with three repetitions and propagated. The concentration measurement is illustrated in Figure 1.

##### 2.1.1.2 Structural Assessment

Structural homogeneity of PAG was assessed with ^1^H MRI on a 3 T Philips Ingenia CX system (Philips Healthcare, Best, Netherlands) by means of a dual‐echo proton density (PD)/$T_{2}$‐weighted acquisition with the following parameters: FOV/nominal resolution = 240 × 240/1 × 1 mm^2^; slice thickness = 3 mm; no. slices = 60; TE1/TE2/TR = 19/85/3500 ms. Images were acquired with a 32‐channel head coil of 5 samples (samples 2–6; see Table 2) held in a custom‐made holder, oriented upright, such that the vial axes were perpendicular to the magnet z‐axis, and image slices showed circular cross‐sections of the phantoms.

**TABLE 2:** A summary of prepared PAG phantom samples with nominal and apparent ion concentrations, showing propagated uncertainties of measurement.

Sample	Nominal [NaCl] (mM)	Apparent [NaCl] (mM)	Nominal [NiCl_2_] (mM)	Apparent [NiCl_2_] (mM)
1	10	9.42 ± 0.29	0.30	0.28 ± 0.01
2	25	23.73 ± 0.43	0.75	0.71 ± 0.01
3	50	46.95 ± 0.74	1.50	1.41 ± 0.02
4	75	72.65 ± 1.09	2.25	2.18 ± 0.03
5	100	97.14 ± 1.50	3.00	2.91 ± 0.04
6	125	116.85 ± 1.78	3.75	3.51 ± 0.04
7	150	140.79 ± 2.11	4.50	4.22 ± 0.06

### 2.2 Relaxometry

#### 2.2.1 Acquisition

All relaxometry acquisitions were performed on a 3 T Philips Ingenia CX system (Philips Healthcare, Best, Netherlands) with a single‐channel birdcage transmit/receive ^23^Na coil (RAPID Biomedical GmbH, Rimpar, Germany). Due to the short $T_{2}$ and rapidly decaying ^23^Na signal, spin echo based methods for $T_{2}$ relaxometry are not feasible. Instead, $T_{2}^{*}$ was measured by directly sampling the FID signal. This was done for each sample, utilising a non‐localised sodium pulse‐acquire sequence with the following parameters: TE = 0.25 ms; RF excitation pulse duration = 320 μs; TR = 200 ms; T_RO_ = 86 ms; BW = 6000 Hz; no. of acquisition samples = 512; number of signal averages (NSA) = 400, FA = 90°. The TE was measured from the centre of the RF pulse to the beginning of the readout. For $T_{1}$ measurement, a 180° inversion pulse was added, with 16 inversion times TI spaced logarithmically from 5 to 120 ms. For all relaxation measurements, each sample was held in a custom‐made holder filled with potassium chloride solution [48] to increase electrical loading of the coil and to provide additional signal for improved ^1^H shimming. The samples were in an upright position (vial axis perpendicular to the magnet z‐axis). Shimming was performed on each sample, utilising a second‐order pencil‐beam volume shim closely encompassing the sample volume. The $T_{2}^{*}$ relaxometry experiment was repeated 6, 12 and 14 months after the first run to monitor phantom stability and probe for any changes in molecular environment.

#### 2.2.2 $T_{2}^{*}$ Fitting

Transverse relaxation of ^23^Na in restricted molecular environments such as tissue, or agarose gel, follows a biexponential relation [3, 49], resulting in the following FID signal:

(1) $$\mathrm{FID}\;\left(t\right)=I_{0}\left[f\exp\left(-\frac{t}{T_{2s}^{*}}\right)+\left(1-f\right)\exp\left(-\frac{t}{T_{2l}^{*}}\right)\right],$$

where $I_{0}$ is the FID amplitude at t=0. If FID signals originate from multiple restricted, complex molecular environments, the observed component fraction, f, may differ from the theoretical value of 0.6 [6]. Fitting for the parameters of the biexponential signal model directly (e.g., by performing a least squares fit of Equation 1) is an ill‐defined problem prone to observer bias [50]. Instead, methods have been developed that estimate the amplitudes of a spectrum, $g\left(\tau \right)$, of predefined $T_{2}^{*}$ values, τ:

(2) $$\mathrm{FID}\left(t\right)=\sum_{\tau =\tau_{\min}}^{\tau_{\max}}g\left(\tau \right)\exp\left(-\frac{t}{\tau }\right).$$

This approach, referred to as inverse Laplace transform, is commonly used in myelin water imaging [51] and has been demonstrated also for $T_{2}^{*}$ fitting of multi‐echo ^23^Na MRI [52]. As in Riemer et al. [52], a MATLAB (The MathWorks, Natick, MA, USA) implementation [53] of Provencher's CONTIN algorithm [54] was utilised.

**FIGURE 3:**
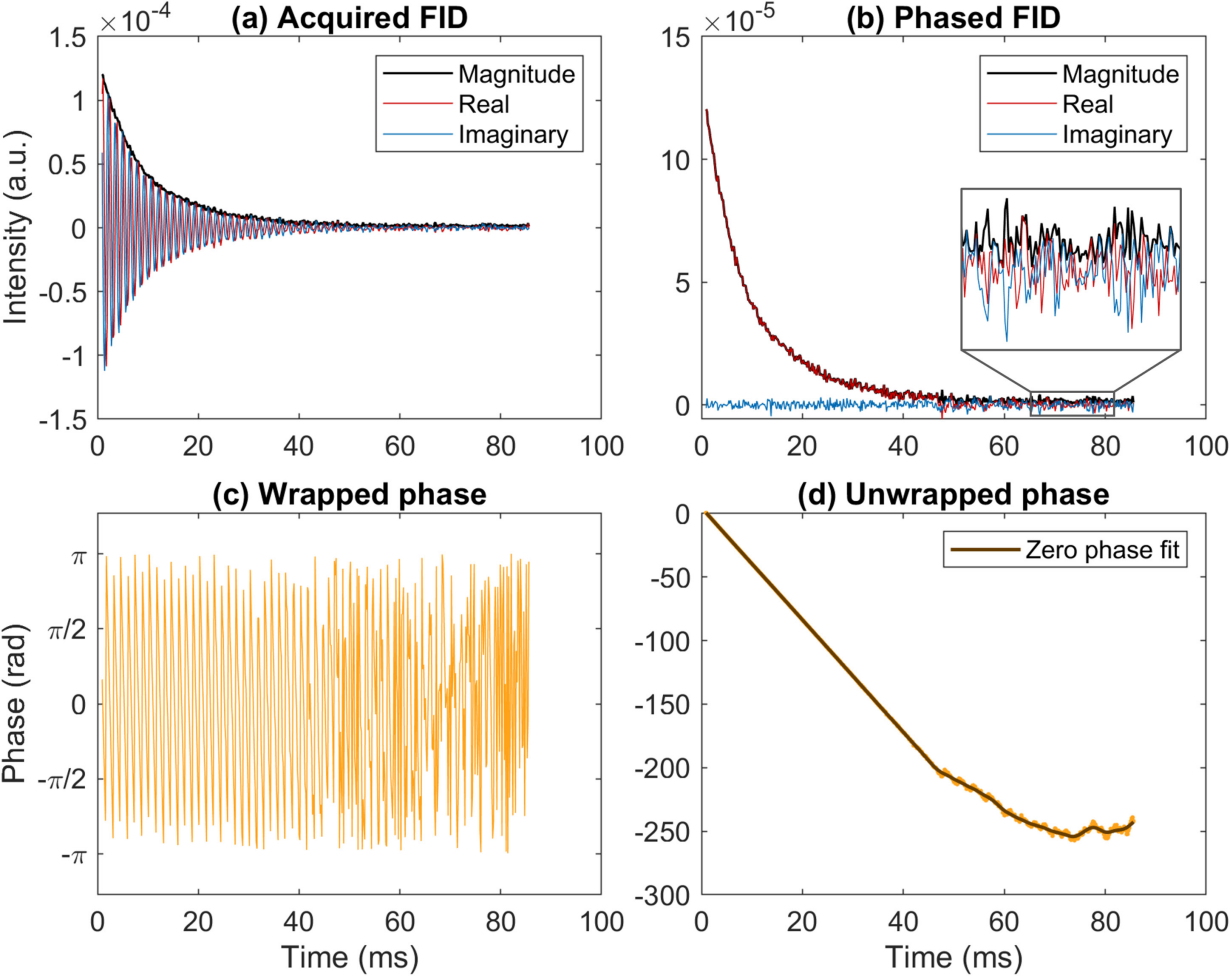
An overview of the processing of complex FID data. In (a), magnitude, real and imaginary parts of the FID are displayed as acquired. The phase is shown in (c) and (d), before and after unwrapping, respectively. The fitted zero‐phase line in (d) is used to derive a complex conjugate exponential term to multiply the complex signal in (a) with, giving rise to the phased FID in (b). During signal decay, the real component closely follows the magnitude; once the signal has decayed, the real part oscillates across the x‐axis, whereas the magnitude oscillates across a positive, non‐zero noise floor, as shown in the zoomed inset. The imaginary part only contains noise.

$T_{2}^{*}$ fitting of time‐truncated magnitude FID data may result in biased $T_{2}^{*}$ estimates due to the positive, non‐zero Rician noise floor, particularly at low SNR conditions [55]. To avoid this bias, the phase of the complex FID was set to zero, and only the real part of the signal was analysed [55]. This was implemented by fitting a smoothed spline through the unwrapped phase and utilising it to estimate a complex conjugate for multiplication. This procedure is illustrated in Figure 3. The first four timepoints of the FID were discarded to remove contamination from RF ring down [12].

For τ, a distribution of 100 linearly spaced $T_{2}^{*}$ values was chosen, ranging from 1 to 50 ms. The fit regularisation strength was set to 0.3. After fitting, the resulting $T_{2}^{*}$ amplitude spectrum, $g\left(\tau \right)$, was processed and interpreted as follows: (i) The number of spectrum peaks was found and related to the exponential decay mode (e.g., two peaks signifies biexponential relaxation); (ii) the peak locations were found and related to the most prominent $T_{2}^{*}$ per decay component; (iii) the relative area under each peak was computed by numerical integration and related to the overall respective contribution per decay component (i.e., f was evaluated by dividing the area under the first peak by the total area); (iv) the peak half width at half maximum was measured to report on the range of $T_{2}^{*}$ present in each decay component, although this exhibits dependence on the regularisation strength. The fitting method was validated with synthetic data (see Table S1 and Figure S1).

#### 2.2.3 $T_{1}$ Fitting

Inversion recovery data were fitted for a monoexponential $T_{1}$ with the following equation using the lsqcurvefit utility in MATLAB (The MathWorks, Natick, MA, USA):

(3) $$M_{z}=M_{0}\left[1-A\exp\left(-\frac{\mathrm{TI}}{T_{1}}\right)\right].$$

An additional parameter, A, was included to account for imperfections in flip angle and power optimisation, likely to occur when scanning individual 50‐mL samples [56]. Due to the relatively short $T_{1}$ of ^23^Na, the TR was estimated to be large enough for the signal recovery between excitation pulses to be complete.

### 2.3 In Vivo Imaging

#### 2.3.1 In Vivo Acquisition and Reconstruction: Brain

^23^Na MRI of a healthy volunteer (F, 24y, written informed consent provided) was performed on a 3 T Philips Ingenia CX system (Philips Healthcare, Best, Netherlands) with a single‐channel birdcage transmit/receive ^23^Na coil (RAPID Biomedical GmbH, Rimpar, Germany). Images were acquired with a 3D cone sequence [57, 58] using the following parameters: FOV/nominal resolution = 240 × 240 × 240/4 × 4 × 4 mm^3^; RF excitation pulse duration = 320 μs; TE/TR = 0.38/120 ms; T_RO_ = 5.6 ms; FA = 90°; NSA = 4. Four quantification phantoms of nominal concentrations from 25 to 100 mM (samples 2–5) were included within the FOV. The TE was measured from the centre of the RF pulse to the beginning of the readout. Automatic shimming was performed over the image volume. Images were reconstructed offline with an in‐house pipeline [58] comprising algorithms for sampling density compensation [59] and a non‐uniform Fourier transform (NUFFT) [60].

#### 2.3.2 In Vivo Acquisition and Reconstruction: Leg Muscle

^23^Na MRI of a healthy volunteer (F, 28y, written informed consent provided) was performed on a 3 T Siemens Biograph mMR system (Siemens Healthineers, Erlangen, Germany). A single‐channel birdcage transmit/receive ^23^Na coil (Stark Contrast, Erlangen, Germany) was positioned around the right calf. Images were acquired with a 2D uniform‐density centre‐out spiral sodium MRI sequence and a half‐sinc excitation pulse [61]. Sequence parameters were as follows: FOV/nominal resolution = 180 × 180/2.25 × 2.25 mm^2^; slice thickness = 30 mm; RF excitation pulse duration = 300 μs; TE/TR = 0.23/100 ms; T_RO_ = 7.6 ms; 20 regularly spaced spiral interleaves and NSA = 150. Gridding reconstruction was performed offline using a NUFFT [62]. Calibration phantoms with nominal sodium concentrations from 10 to 100 mM (samples 1–5) were rested on top of the leg, within the coil and image FOV. Manual B_0_ shimming and a global flip angle calibration were performed.

#### 2.3.3 aTSC Quantification

Phantoms were masked manually and mean intensities were calculated. A linear calibration curve was fitted between the mean intensities (y‐axis) and measured apparent phantom sodium concentrations (x‐axis). Raw images were brain‐ or leg‐masked and scaled to represent aTSC, according to the linear fit parameters, producing conventional aTSC maps.

To quantify the measurement uncertainty of aTSC results, a parametric bootstrap was applied. This involved resampling the data points of the calibration curve from their respective probability distributions: in x: a uniform distribution bounded by the errors of measurement of sodium concentrations, in mM (see Table 2); and in y: a normal distribution parametrised by the mean and variance calculated from masked phantom ROIs. This was repeated over 1000 iterations, after which mean and standard deviation aTSC maps were calculated.

Results for the above quantification procedures were reported in anatomical ROIs of brain white matter (WM) and leg muscle (see Supporting Information S2 and Figure S2 for further details).

## 3 Results

### 3.1 Phantom Preparation

Table 2 summarises the nominal and apparent concentrations of NaCl and NiCl_2_ in all seven PAG samples, as well as their uncertainties propagated from errors of measurement. Apparent concentrations were 93%–97% of nominal ones.

**FIGURE 4:**
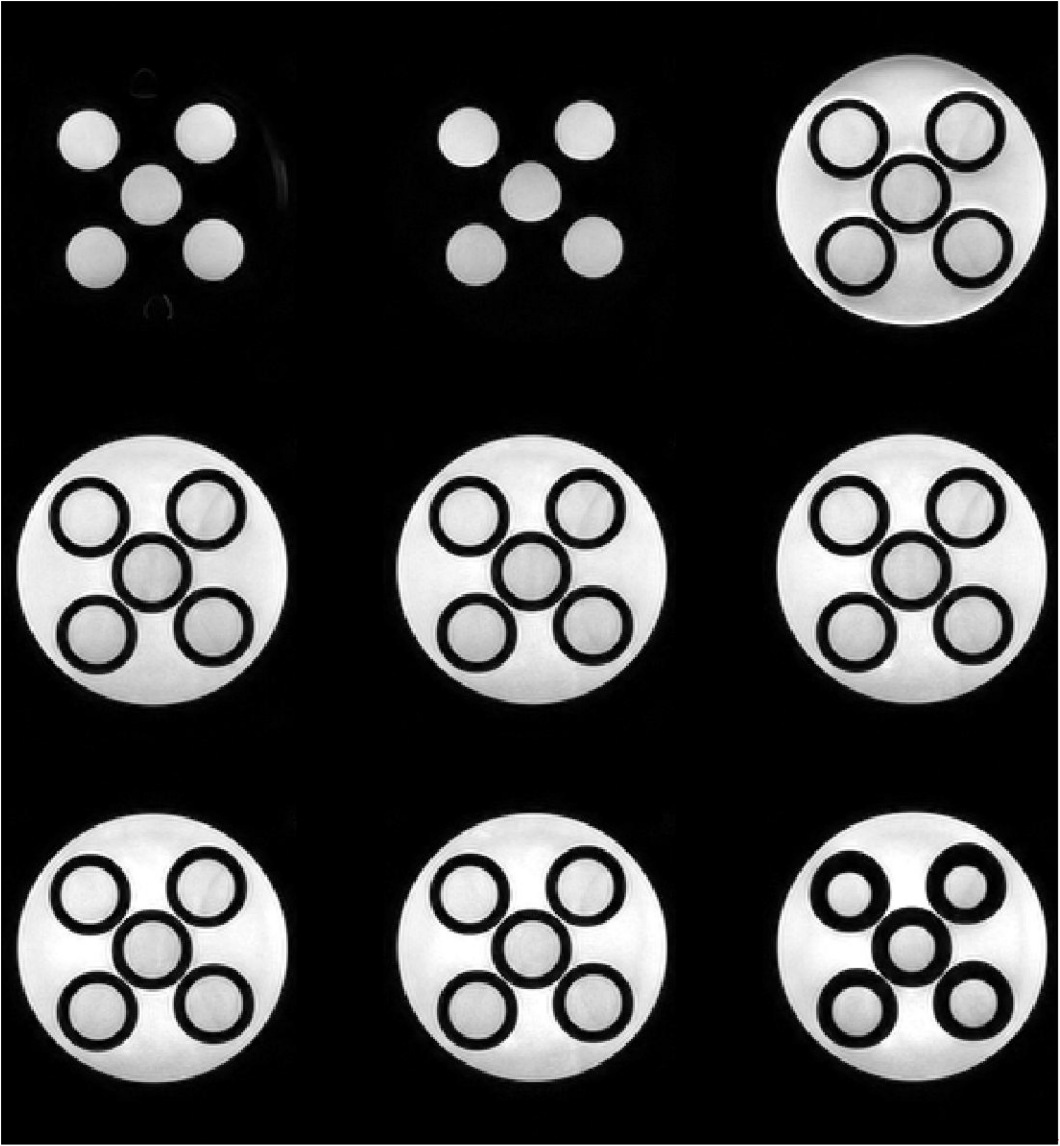
Nine different slices (XZ plane) of five polyacrylamide gel (PAG) phantom vials (samples 2–6, nominal [NaCl] of 25–125 mM and [NiCl_2_] of 0.75–3.75 mM) acquired with a proton density (PD) weighted ^1^H MRI scan to ascertain structural homogeneity. Phantoms are in a holder containing potassium chloride solution. Signal in the phantoms appears uniform, with no evidence of bubbles, macroscopic compartmentalisation or other unexpected patterns in image intensity. Diffuse Nyquist ghosting of the potassium chloride solution is visible in the rightmost phantoms, vanishing in the first two slices where the vials protrude from the holder.

Figure 4 shows PD‐weighted ^1^H MRI images of five PAG vials (samples 2–6) acquired to inspect structural homogeneity. The phantoms exhibit homogeneous signal throughout, without any evidence of macroscopic compartmentalisation or imperfections, such as cracks or air bubbles.

### 3.2 Relaxometry

**FIGURE 5:**
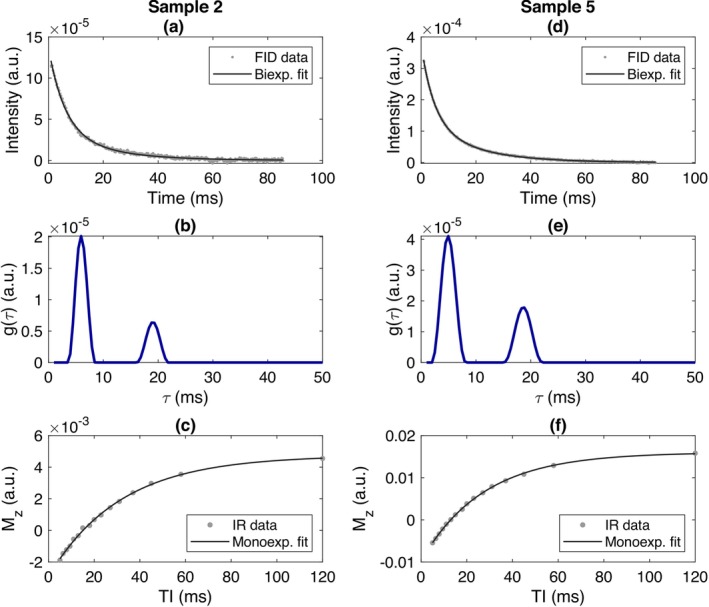
Relaxometry data and fitted curves for samples 2 (a–c) and 5 (e–f), giving examples at both low and high sodium concentration regimes. Plots (a) and (d) show data (grey points) and fitted biexponential relationships (black line, Equation 1) of the FID signal to measure the $T_{2}^{*}$ of phantoms. Plots (b) and (e) show the corresponding spectra of fitted $T_{2}^{*}$ values ($g\left(\tau \right)$ in Equation 2). The two peaks of the spectra clearly indicate a biexponential decay mode. Plots (c) and (f) show data (grey points) and fits of Equation (3) (black line) for an inversion recovery experiment to measure the $T_{1}$.

Inversion recovery and FID data, along with fitted curves, are shown in Figure 5 for samples 2 and 5 (plots of all samples are in Figure S3). Table 3 contains all quantitative relaxometry data (longitudinal, biexponential $T_{2}^{*}$ and monoexponential $T_{1}$) for all seven PAG samples and the agarose gel sample; this is also reproduced visually as a bar chart in Figure S4.

**TABLE 3:** All quantitative relaxometry results for polyacrylamide gel (PAG) phantom samples, as well as an agarose sample.

Sample 1: 10 mM							
Repeat	$T_{2s}^{*}$ (ms)	±	$T_{2l}^{*}$ (ms)	±	f	$T_{1}$ (ms)	±
1 (Month 0)	6.9	1.2	18.8	1.3	0.80	39.0	3.9
2 (Month 6)	6.4	1.3	13.4	1.6	0.55		
3 (Month 12)	8.4	1.4	23.8	1.3	0.96		
4 (Month 14)	6.4	1.3	17.3	1.5	0.76		
**Mean/SD**	**7.1/0.9**		**18.3/4.3**		**0.77/0.17**		
Average change	−0.2		−0.5		−0.01		
Coeff. of variation (%)	13.3		23.4		22.0		

Sample 2: 25 mM							
Repeat	$T_{2s}^{*}$ (ms)	±	$T_{2l}^{*}$ (ms)	±	f	$T_{1}$ (ms)	±
1 (Month 0)	5.9	1.2	18.8	1.4	0.73	31.5	2.2
2 (Month 6)	5.0	1.2	13.4	1.5	0.49		
3 (Month 12)	5.9	1.5	18.3	1.6	0.78		
4 (Month 14)	5.5	1.3	17.3	1.5	0.73		
**Mean/SD**	**5.6/0.5**		**17/2.5**		**0.68/0.13**		
Average change	−0.2		−0.5		0.00		
Coeff. of variation (%)	8.5		14.6		19.1		

Sample 3: 50 mM							
Repeat	$T_{2s}^{*}$ (ms)	±	$T_{2l}^{*}$ (ms)	±	f	$T_{1}$ (ms)	±
1 (Month 0)	5.5	1.2	18.8	1.5	0.70	28.1	1.2
2 (Month 6)	5.5	1.5	16.8	1.7	0.64		
3 (Month 12)	6.4	1.4	19.3	1.5	0.75		
4 (Month 14)	5.5	1.3	17.3	1.6	0.68		
**Mean/SD**	**5.7/0.5**		**18.1/1.2**		**0.69/0.05**		
Average change	0.0		−0.5		−0.01		
Coeff. of variation (%)	8.7		6.5		6.6		

Sample 4: 75 mM							
Repeat	$T_{2s}^{*}$ (ms)	±	$T_{2l}^{*}$ (ms)	±	f	$T_{1}$ (ms)	±
1 (Month 0)	4.5	1.2	17.3	1.5	0.64	27.5	0.9
2 (Month 6)	4.5	1.4	15.4	1.6	0.60		
3 (Month 12)	5.5	1.3	17.8	1.6	0.67		
4 (Month 14)	5.0	1.3	16.8	1.5	0.64		
**Mean/SD**	**4.8/0.5**		**16.8/1.1**		**0.64/0.03**		
Average change	0.2		−0.2		0.00		
Coeff. of variation (%)	9.8		6.3		4.5		

Sample 5: 100 mM							
Repeat	$T_{2s}^{*}$ (ms)	±	$T_{2l}^{*}$ (ms)	±	f	$T_{1}$ (ms)	±
1 (Month 0)	5.0	1.5	18.8	1.7	0.67	27.3	0.9
2 (Month 6)	5.0	1.6	17.3	1.8	0.63		
3 (Month 12)	5.9	1.6	20.8	1.8	0.73		
4 (Month 14)	5.0	1.3	18.3	1.6	0.67		
**Mean/SD**	**5.2/0.5**		**18.8/1.5**		**0.68/0.04**		
Average change	0.0		−0.2		0.00		
Coeff. of variation (%)	9.5		7.7		6.1		

Sample 6: 125 mM							
Repeat	$T_{2s}^{*}$ (ms)	±	$T_{2l}^{*}$ (ms)	±	f	$T_{1}$ (ms)	±
1 (Month 0)	4.5	1.6	17.8	1.9	0.66	26.9	0.9
2 (Month 6)	4.5	1.4	17.3	1.7	0.64		
3 (Month 12)	5.0	1.3	19.3	1.6	0.68		
4 (Month 14)	5.0	1.3	18.3	1.6	0.65		
**Mean/SD**	**4.7/0.3**		**18.2/0.8**		**0.66/0.02**		
Average change	0.2		0.2		0.00		
Coeff. of variation (%)	6.1		4.6		2.6		

Sample 7: 150 mM							
Repeat	$T_{2s}^{*}$ (ms)	±	$T_{2l}^{*}$ (ms)	±	f	$T_{1}$ (ms)	±
1 (Month 0)	4.5	1.4	17.8	1.7	0.65	26.7	0.9
2 (Month 6)	4.5	1.3	17.3	1.6	0.64		
3 (Month 12)	5.0	1.3	18.8	1.7	0.65		
4 (Month 14)	5.0	1.3	17.8	1.7	0.65		
**Mean/SD**	**4.7/0.3**		**18/0.6**		**0.65/0.01**		
Average change	0.2		0.0		0.00		
Coeff. of variation (%)	6.1		3.5		0.8		

Agarose control: 85 mM							
Repeat	$T_{2s}^{*}$ (ms)	±	$T_{2l}^{*}$ (ms)	±	f	$T_{1}$ (ms)	±
1 (Month 6)	5.0	1.3	17.8	1.5	0.63	37.7	0.8
2 (Month 12)	5.5	1.5	18.3	2.0	0.66		
3 (Month 14)	5.5	1.5	18.3	1.8	0.63		
**Mean/SD**	**5.3/0.3**		**18.2/0.3**		**0.64/0.02**		
Average change	0.2		0.2		0.00		
Coeff. of variation (%)	5.4		1.6		2.7		

*Note:* Sodium concentrations indicated are nominal. Biexponential $T_{2}^{*}$ values reported are the peak locations of fitted $T_{2}^{*}$ spectra; peak half‐widths are indicated as ± bounds. For longitudinal data, summary statistics include a mean and standard deviation of all repeats, an average change (the mean of the differences between consecutive repeats) and the coefficient of variation (standard deviation divided by mean). The ± bounds for $T_{1}$ values indicate the standard error of the fit.

Relaxation parameters in PAG were found to range from $T_{2s}^{*}$ = 4.7–7.1 ms, $T_{2l}^{*}$ = 16.8–18.8 ms, short component fraction f = 0.64–0.77, $T_{1}$ = 27–39 ms. In the agarose reference, average relaxation parameters were $T_{2s}^{*}$ = 5.3 ± 0.3 ms, $T_{2l}^{*}$ = 18.2 ± 0.3 ms, short component fraction f = 0.64 ± 0.02, $T_{1}$ = 37.7 ± 0.8 ms. Parameters for PAG sample 1 (10 mM nominal sodium concentration) appear to be outliers, especially $T_{1}$. Across longitudinal scans, the absolute average change of $T_{2}^{*}$ is less than 0.5 ms for all samples and approximately an order of magnitude smaller than the reported range of $T_{2}^{*}$ per component peak. The coefficient of variation of relaxation parameters was below 10% for samples 3–7 (50–150 mM), below 20% for sample 2 (25 mM) and up to 23% for sample 1 (10 mM).

### 3.3 In Vivo ^23^Na MRI

**FIGURE 6:**
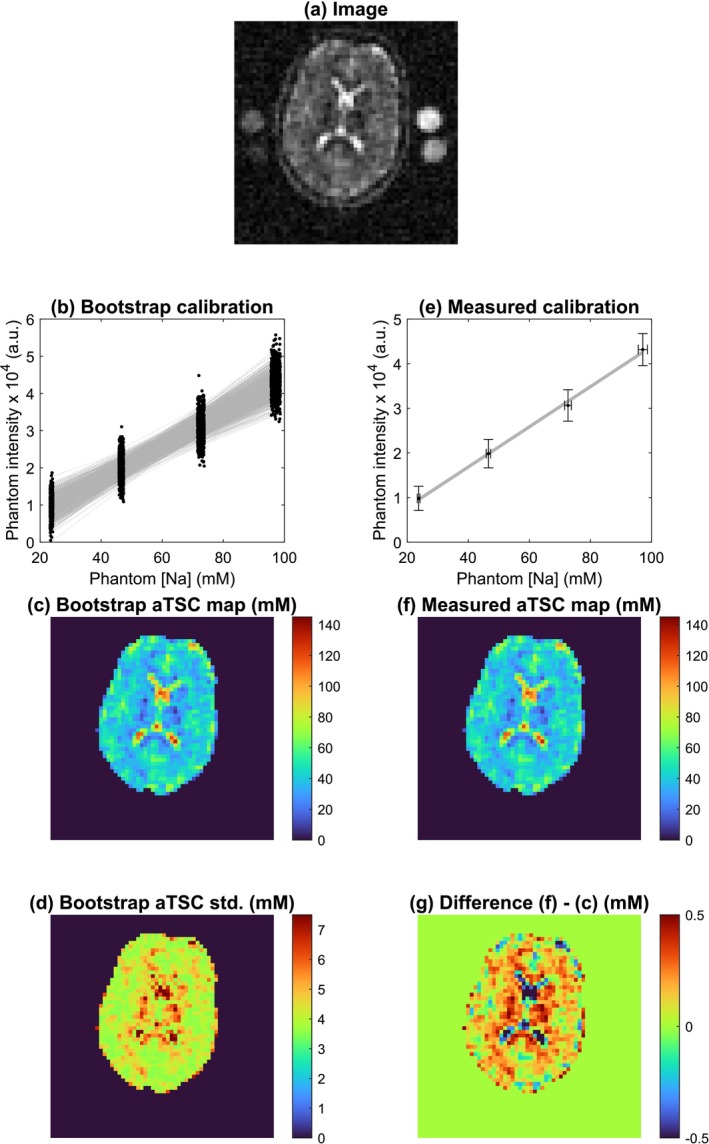
Results of in vivo ^23^Na MRI of the human brain. The raw intensity image with calibration phantoms is shown in (a). A conventional quantification procedure with calibration curve and resulting aTSC map is shown in (e) and (f). A bootstrap quantification procedure with 1000 iterations, along with the resulting mean and standard deviation aTSC map, is shown in (b), (c) and (d). Finally, (g) shows the difference between (f) and (c). Note different intensity scales in (d) and (g).

**FIGURE 7:**
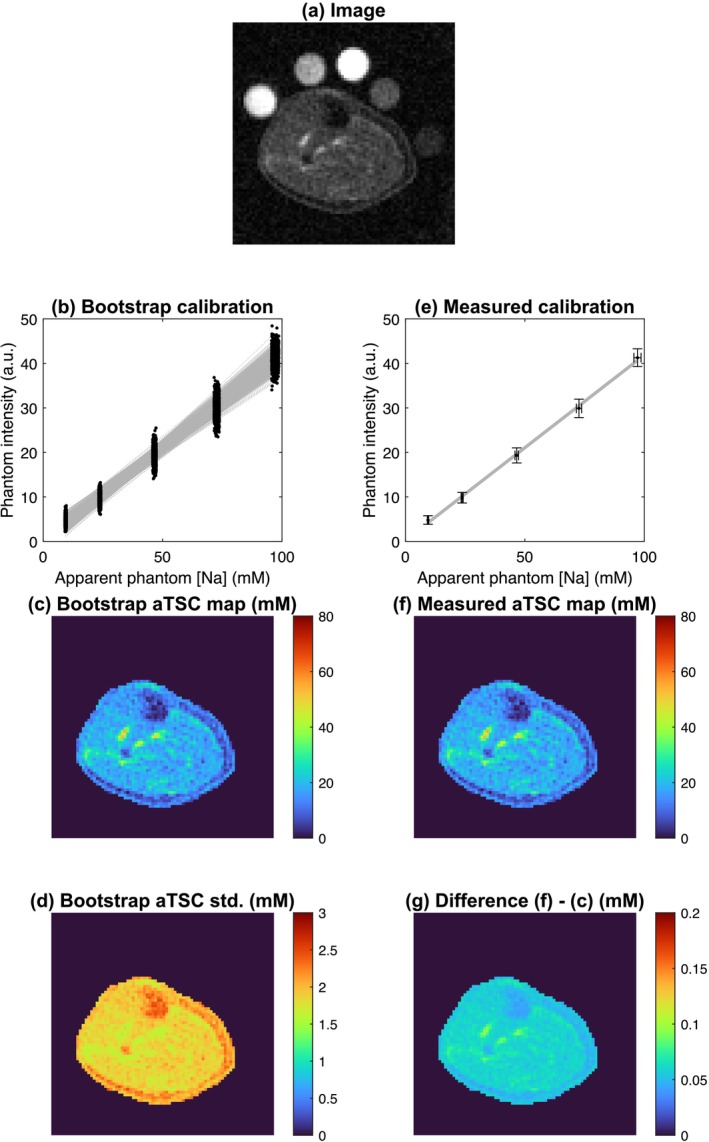
Results of in vivo ^23^Na MRI of human calf muscle. The raw intensity image with calibration phantoms is shown in (a). A conventional quantification procedure with calibration curve and resulting aTSC map is shown in (e) and (f). A bootstrap quantification procedure with 1000 iterations, along with the resulting mean and standard deviation aTSC map, is shown in (b), (c) and (d). Finally, (g) shows the difference between (f) and (c).

Figures 6 and 7 show acquired in vivo ^23^Na MRI images of the human brain and calf muscle, respectively. They also show reconstructed quantitative aTSC maps, comparing a conventional quantification approach (simple linear fit) with the mean aTSC map from a bootstrapping approach. By visual assessment, aTSC ranges between approximately 20–50 mM in brain tissue and 100–130 mM in CSF; in leg muscle, aTSC ranges between approximately 15 and 30 mM. In a brain WM ROI, aTSCs of 31.9 ± 7.6 mM and 31.7 ± 7.5 mM were found for measured and bootstrap‐derived maps, corresponding to a respective mean difference of 0.3 ± 0.1 mM. The bootstrap‐derived voxel‐wise standard deviation in the WM ROI was 4.8 ± 0.7 mM. In a leg muscle ROI, aTSCs of 18.1 ± 3.4 mM and 18.1 ± 3.4 mM were found for measured and bootstrap‐derived maps, corresponding to a respective mean difference of 0.1 ± 0.0 mM. The bootstrap‐derived voxel‐wise standard deviation in the WM ROI was 1.9 ± 0.1 mM.

## 4 Discussion

We successfully demonstrated the use of PAG for producing calibration phantoms with different sodium concentrations for quantitative in vivo ^23^Na MRI. As we discuss below, these synthetic phantoms are an important step towards standardisation of quantification using ^23^Na MRI.

### 4.1 Phantom Assessment

#### 4.1.1 Preparation of Cross‐Linked PAG

PAG is a synthetic polymer hydrogel, formed by the polymerisation reaction of acrylamide monomers. Polyacrylamide can be synthesised with a cross‐linking agent, such as *N,N′*‐methylenebisacrylamide (bis‐acrylamide), as in this study. Cross‐linkers make connections between polyacrylamide chains in the form of covalent bonds, yielding a more complex molecular structure than linear arrangements of polymer strands. Many simplified representations [63] consider it an irregular mesh of polymer strands and cross‐linking sites. However, Gombert et al. provide an excellent account of the true complexity of PAG's molecular structure, looking “beyond the fishing net” [64]. In fact, the molecular structure of PAG can vary greatly with concentrations of monomer and cross‐linker. Gombert et al. describe different structural regimes, which also manifest in different physical appearances, ranging from transparent to cloudy and milky‐white opaque (as the PAG samples prepared in this study) with an increasing proportion of cross‐linker. For the latter, cross‐linkers are described to agglomerate into μm‐sized domains that, visually represented [64], mimic intracellular structures surrounded by an extracellular matrix.

Each sample was prepared by diluting a specific quantity of highly concentrated stock solution with a solution containing polyacrylamide monomers. As the density of the latter was unknown and no volumetric measurements were carried out during preparation, the actual molarity sodium concentration of each sample could not be evaluated accurately. Therefore, an apparent sodium concentration of samples was estimated volumetrically after preparation by means of a high precision scale and inert oil of known density. On average, the apparent sodium concentrations were 5% lower compared to nominal ones. This reflects both the contribution of the solid volume fraction to the apparent concentration, as well as gel expansion during synthesis (Gombert et al. describe gel swelling [64]). An advantage of PAG over agarose is that synthesis is possible at room temperature and reagents need not be heated at any stage. Therefore, discrepancies between actual and nominal sodium concentrations due to evaporation during sample preparation will be negligible. In the future, the molarity sodium concentration of samples should also be evaluated accurately, either by modifying the preparation procedure or by performing additional molarity measurements during preparation.

#### 4.1.2 Relaxation Parameters

The fitted relaxation parameters of PAG are within or close to the ranges of those measured in vivo ($T_{2s}$ = 0.5–5.5 ms, $T_{2l}$ = 15–40 ms, f ≈ 0.6, $T_{1}$ = 15–35 ms) [1, 2, 4]. The short component fraction, f, is slightly higher than 0.6 in PAG, although in vivo measurements of f are uncommon, as f = 0.6 is often used as constant for in vivo relaxometry [4, 40]. Also, fitted relaxation parameters of the agarose reference sample are in agreement with past reports on agarose [5, 39, 40, 65, 66]. Although biexponential $T_{2}^{*}$ values closely agree between the agarose reference and PAG, PAG was found to have a shorter $T_{1}$, possibly better matching that in vivo [4] (although $T_{1}$ measurements in literature are limited and quite heterogeneous). This means that longitudinal recovery is mostly complete at typically used TR = 120 ms, such that $T_{1}$ correction factors previously used with agarose [15] may not be necessary. Altogether, relaxometry suggests that accuracy of aTSC quantification with PAG phantoms should not be subject to significant relaxation bias. For applications where shorter $T_{2s}^{*}$ may be desirable, preparation of PAG at higher gelling agent concentrations and cross‐linker proportions could be explored.

The relaxation parameters of sample 1 were consistently different to those of other samples. Relaxation parameters are not expected to be invariant of sodium concentration. Indeed, such dependencies have recently been simulated in saline [67] and measured in NMR studies of biopolymers [5], as well as in ^23^Na MRI of agarose [40], although concentrations as low as 10 mM are not typically reported. As observed relaxation parameters are the product of a coarse averaging of complex spatiotemporal molecular dynamics, it may be that at particularly low sodium concentrations, this “average” is no longer meaningful (e.g., the molecular interactions that drive biexponential relaxation do not occur frequently enough). Alternatively, it could be a systematic error introduced by discrepancies of SNR, or different B_0_ inhomogeneity conditions across samples, as different NiCl_2_ doping altered the ^1^H signal used for shimming across samples.

Future work should closely investigate the molecular environments of PAG; due to its structural complexity [64], as well as the presence of different types of macromolecules (monomer and cross‐linker), the molecular environment of ^23^Na ions may not be entirely homogeneous, and it is possible that the observed ^23^Na signal is a superposition from multiple compartments. Indeed, PAG samples prepared at lower gelling agent concentrations during an exploratory part of the study (see Table S5) deviate from the theoretical f = 0.6, as expected in a homogeneous substance with a restricted molecular environment [3, 6]. Gombert et al. describe that PAGs with lower proportions of cross‐linker undergo greater swelling, forming “pockets” of water; it is possible that at certain pocket sizes, ^23^Na ions also encounter a free water environment. Alternatively, there could be residual quadrupolar interactions due to ordered macromolecular structures, which are known to affect relaxation [13], but due to the random nature of the polymer network, this may be less likely. The presence of a residual quadrupolar interaction could be probed by conducting further studies with pulse sequences that may be sensitive to molecular environment, such as multiple quantum filtering (MQF) [68]. This could also explore the suitability of PAG phantoms for in vivo MQF studies [69]. Although this study focused on testing and demonstrating PAG phantoms on more widely available, clinical 3 T systems, ^23^Na MRI is increasingly performed at ultra‐high fields. Future work should therefore also consider assessing the relaxation properties of PAG phantoms at 7 T or above, given their dependence on field strength [41].

#### 4.1.3 Stability

Considering the average change of biexponential $T_{2}^{*}$ reported in Table 3, estimated parameters are deemed constant over 14 months, within their coefficients of variation (below 10% for most parameters and samples). This is a highly encouraging result regarding the stability and longevity of PAG sodium quantification phantoms and their potential application in long‐term or longitudinal cohort studies. It is implied that any kind of significant changes in the molecular environment would reflect in systematic deviations to observed $T_{2}^{*}$ parameters. Woessner reported structural alterations with time in gelatine and xanthan gum by studying FID shape, also noting that $T_{1}$ was not sensitive to such alterations [5]. It is not clear whether relaxometry would be sensitive to evaporation processes that may cause a gradual drift in the apparent sodium concentration of a phantom. However, a scientific‐grade vial with an airtight seal should sufficiently restrict evaporation, though phantoms could be double‐sealed with, for example, wax. Ongoing data acquisition will confirm stability and allow calculation of statistical significance.

Although the non‐localised pulse‐acquire approach samples the FID at SNR and temporal resolution far superior than obtainable through imaging, it also has limitations. The $T_{2}^{*}$ parameter may not be the most suitable metric for assessment of stability, due to its inherent dependence on field inhomogeneities, and therefore experimental conditions, such as set‐up, sample geometry and shimming. The non‐localised acquisition scheme may be particularly sensitive to the aforementioned; for example, susceptibility effects from the air–sample interface will cause global, not local signal artefacts, although the fitting method partially accounts for this by modelling a spectrum of $T_{2}^{*}$ values. Field inhomogeneities and shimming are likely to be the main source of some heterogeneity in $T_{2}^{*}$ estimates across samples and repeats, considering, for example, anomalous parameters in repeat 2 for samples 1 and 2. Our advice is therefore to acquire a ^1^H B_0_ map to inspect shim quality, which was done ad hoc for illustrative purposes on a single sample (see Figure S6).

Synthetic polymer hydrogels, such as polyacrylamide, by default exhibit desirable properties for standardisation (e.g., consistency, stability and lifespan) compared to biopolymer gels like agarose. Nonetheless, polyacrylamide can still be prone to degradation by depolymerisation, though the vulnerability to such processes will depend strongly on the application environment. Degradation can be split into thermal, biological, chemical, mechanical and photolytic mechanisms [70]. Sealed MRI phantoms are highly unlikely to undergo to biological or chemical degradation or encounter temperatures close to or above the critical threshold for thermal degradation [70]. Photodegradation only occurs at infrared, visible and UV wavelengths corresponding to the required photon energies for polymer bond dissociation [70]. As long as phantoms are adequately stored, away from incident sunlight, this is unlikely to be an issue. Lastly, it may be that vibrations or simple ‘wear and tear’ could cause some mechanical degradation, but as PAG exhibits some elasticity and can be made less brittle than agarose, resistance to mechanical stress (e.g., accidental drops from height) may be improved. Altogether, we are confident that there are no obvious limits to the lifespan of PAG phantoms. As there is a significant temperature dependence of sodium relaxation parameters [71], we advise against refrigerating the phantoms, instead storing them at ambient temperature in the scanner room.

A limitation of this study is that PAG phantoms were not directly compared to a corresponding set of agarose quantification phantoms. This choice was made with the a priori assumption that a synthetic gel phantom would be of greater value to the community. Measurements of relaxation parameters were still made on an individual agarose phantom, which also remained stable over time. Although agarose phantoms are inherently more prone to deterioration (e.g., by cracking or formation of mould [4, 44]), we acknowledge that, when prepared carefully, to a high standard, they may still provide a stable quantification phantom.

### 4.2 In Vivo ^23^Na MRI

In vivo aTSC values quantified with PAG phantoms, shown in Figures 6 and 7, lie well within literature reported ranges [1, 4], both in the human brain and human calf muscle. Nonetheless, reported aTSC carry a discrepancy relating to the solid volume fraction, as the measured *apparent* sodium concentrations of phantoms were utilised for quantification. For a truly accurate TSC measurement, aTSC values should be multiplied by the tissue WF. For instance, Christensen et al. applied this scaling step when using saline calibration standards utilising an experimentally determined whole brain WF of 0.82 (ex vivo laboratory analysis in rats) [72]. Although perhaps more accurate, such a global rescaling fails to account for regional differences in WF of brain or leg tissue and was therefore not applied here. An improvement may be to apply tissue‐wise WFs from literature (0.7 for white matter and 0.85 for grey matter [8]), following Madelin et al [9]. If a TSC measurement is to be as accurate as possible, it should include a voxel‐wise measurement of the WF, for example, with quantitative proton density mapping methods [73, 74, 75]. This could be informative also in case pathological changes alter the WF as well as sodium physiology.

Here, we also present maps of the uncertainty introduced by quantification (i.e., associated to the calibration curve, not the entire MR measurement process); this is reported as the voxel‐wise standard deviation following a 1000 iteration bootstrap quantification. The standard deviation is higher in regions of more extreme aTSC (e.g., CSF or deep grey matter), where the resampled calibration curves fan out as shown in Figures 6b and 7b. It also differs between brain and leg scans, which is not surprising considering the different hardware, sequences and sampling approaches. We believe that calculating the standard deviation as proposed here is important, especially during study design, such as for prospective power calculations, or for selecting phantoms. For example, the number of phantoms and their sodium concentrations could be chosen to minimise the standard deviation of aTSC in the range of values corresponding to the target tissue.

It should be noted that the standard deviation of signal in phantom ROIs, used to parametrise the normal distribution from which calibration points are resampled, is not a fair representation of the uncertainty in signal intensity. For instance, the chosen reconstruction method will certainly influence the apparent standard deviation of the signal, as greater oversampling factors for gridding, post‐acquisition filtering and inverse problem or compressed sensing reconstruction approaches may all yield smoother images, without truly improving precision. The standard deviation also fails to account for quantification errors due to differences in relaxation parameters. A possible subject of further investigation in the context of more precise in vivo quantification methods may be to extend the bootstrap approach to incorporate TR, TE and ranges of $T_{1}$, $T_{2}^{*}$ when resampling points, accounting also for residual relaxation weighting.

Finally, it is important to state that quantification phantoms are one of many factors that could lead to variability or errors in TSC measurements. Partial volume effects, different acquisition schemes and residual relaxation weightings or B_1_ inhomogeneities and positioning of phantoms, among others, could all play a role. A dedicated study would be needed to uncover the most prominent factors that drive TSC variability, as well as the precise contribution of quantification phantoms. A solution for the consistent positioning of calibration phantoms may also be found by future work, perhaps incorporating a phantom support structure into coil designs, particularly for surface coils with inhomogeneous B_1_^+^, or for modern multi‐channel coils with little available space [37].

## 5 Conclusion

In conclusion, we demonstrated the preparation of novel PAG phantoms for ^23^Na MRI and their use for aTSC quantification in brain tissue and calf muscle. Results show that PAG yields the desired characteristics of a ^23^Na MRI phantom: favourable relaxation properties for accurate measurements, structural homogeneity and stability (both intrinsic and of relaxation parameters) with time and solid (gel) state of matter.

As a synthetic polymer substance, PAG is inherently more traceable and suitable for standardisation than naturally derived alternatives. Further, we could not identify any apparent drawbacks of using PAG phantoms for aTSC quantification. Amongst other factors, we believe that standardised quantification phantoms are a necessary step towards harmonisation of methods and achieving more concordant aTSC measurements across centres and sites. Indeed, PAG phantoms have already been distributed to additional sites, making multicentre studies on PAG phantoms a future possibility.

## Supporting information


**Figure S1.** To verify accuracy of the $T_{2}^{*}$ fitting method, a biexponentially decaying signal was simulated with: $T_{2s}^{*}$ = 3 ms, $T_{2l}^{*}$ = 18 ms, f = 0.6 ms and additive complex Gaussian noise of zero mean and standard deviation σ = [0.001, 0.01], simulating both ‘low SNR’ and ‘high SNR’ regimes. Sequence parameters (TE, bandwidth and no. of samples) were chosen in accordance with the acquisition protocol. Fitting was performed on the real component and the magnitude of the signal. Above, the first row of plots show the simulated signals and fitted biexponential decays; the second row of plots show the fitted $T_{2}^{*}$ spectra; columns show different SNR levels. The importance of utilising the real signal component is illustrated, as magnitude data induce a bias in $T_{2l}^{*}$ estimates at lower SNR (and therefore sodium concentrations).
**Table S1.** This table summarises the numerical results of the simulation experiment described in Figure S1. Fitted parameters are in good agreement with theoretical ones ($T_{2s}^{*}$ = 3 ms, $T_{2l}^{*}$ = 18 ms, f = 0.6 ms), apart from the case of fitting on magnitude data at low SNR. Results imply the fitting method is of good accuracy.
**Figure S2.** Overlays of the analysis ROIs utilised for aTSC calculations on ^23^Na MRI images of the human brain (left) and human calf muscle (right). For the brain, a white matter specific ROI of 107 voxels was derived by applying an automatic segmentation algorithm (SynthSeg, https://github.com/BBillot/SynthSeg ^i^) to an additionally acquired T_2_‐weighted ^1^H scan in the same image space. The resulting probabilistic segmentation was resampled and thresholded at 0.95 likelihood for white matter. For the leg, a conservative ROI of 366 voxels was manually drawn inside muscle tissue. ^i^Billot B, Magdamo C, Cheng Y et al. Robust machine learning segmentation for large‐scale analysis of heterogeneous clinical brain MRI datasets. *Proc. Natl. Acad. Sci. U.S.A.* 2023;120 (9) e2216399120, https://doi.org/10.1073/pnas.2216399120.
**Figure S3.** Relaxometry data and fitted curves for all samples 1–7. Each tile of three plots shows the FID signal and biexponential fit (top row), the fitted spectrum of $T_{2}^{*}$ values (middle row) and monoexponential inversion recovery data and fit (bottom row).
**Figure S4.** A visual representation of the biexponential $T_{2}^{*}$ and monoexponential $T_{1}$ relaxometry results for all phantom samples 1–7, as listed in Table 3. Charts (a)–(c) show fitted $T_{2s}^{*}$, $T_{2l}^{*}$ in grey and blue, as well as component ratios, for 4 longitudinal repeats at 0, 6, 12 and 14 months. Error bars indicate the widths of fitted spectrum peaks. Chart (d) shows fitted $T_{1}$. Error bars indicate standard errors of the fit. Bars represent individual measurements.
**Table S5.** For initial, exploratory experiments (otherwise not reported), PAG samples were prepared at different concentrations of gelling agents. This table reports the results for $T_{2}^{*}$ relaxometry of two such samples: X1, prepared with 3% gelling agent, of which 5% was cross‐linker; X2, prepared with 10% gelling agent, of which 5% was cross‐linker. Both were prepared at 85 mM nominal sodium concentration. Fitted biexponential $T_{2}^{*}$ values differ significantly from those found in the prototype samples analysed as part of this study, demonstrating that PAG could be prepared at different concentrations to generate phantoms with unique relaxation properties. The origin of such variations of $T_{2}^{*}$ with PAG concentration should be a subject of further investigation.
**Figure S6.** A masked B_0_ field map showing offsets to the sodium resonance frequency across the volume of a PAG phantom vial, following a second‐order shim routine as applied before non‐selective spectroscopic measurements. Particularly the top of the vial, which protrudes from the sample holder, contains a greater spectrum of precession frequencies. The field map was acquired and reconstructed using a vendor provided B_0_ field mapping protocol (for ^1^H, 1 ms echo spacing, rescaled to the ^23^Na frequency).

## Data Availability

The data that support the findings of this study are available from the corresponding author upon reasonable request.
