# Supplementary material for: Polyacrylamide Gel Calibration Phantoms for Quantification in Sodium MRI
Source: NMR Biomed. 2025 May 7;38(6):e70056. doi: 10.1002/nbm.70056 (PMC12056480; doi:10.1002/nbm.70056)
Supplement: Supplementary file 1 — Figure S1. To verify accuracy of the T2* fitting method, a biexponentially decaying signal was simulated with: T2s* = 3 ms, T2l* = 18 ms, f = 0.6 ms and additive complex Gaussian noise of zero mean and standard deviation σ = [0.001, 0.01], simulating both ‘low SNR’ and ‘high SNR’ regimes. Sequence parameters (TE, bandwidth and no. of samples) were chosen in accordance with the acquisition protocol. Fitting was performed on the real component and the magnitude of the signal. Above, the first row of plots show the simulated signals and fitted biexponential decays; the second row of plots show the fitted T2* spectra; columns show different SNR levels. The importance of utilising the real signal component is illustrated, as magnitude data induce a bias in T2l* estimates at lower SNR (and therefore sodium concentrations). Table S1. This table summarises the numerical results of the simulation experiment described in Figure S1. Fitted parameters are in good agreement with theoretical ones (T2s* = 3 ms, T2l* = 18 ms, f = 0.6 ms), apart from the case of fitting on magnitude data at low SNR. Results imply the fitting method is of good accuracy. Figure S2. Overlays of the analysis ROIs utilised for aTSC calculations on 23Na MRI images of the human brain (left) and human calf muscle (right). For the brain, a white matter specific ROI of 107 voxels was derived by applying an automatic segmentation algorithm (SynthSeg, https://github.com/BBillot/SynthSeg i) to an additionally acquired T2‐weighted 1H scan in the same image space. The resulting probabilistic segmentation was resampled and thresholded at 0.95 likelihood for white matter. For the leg, a conservative ROI of 366 voxels was manually drawn inside muscle tissue. iBillot B, Magdamo C, Cheng Y et al. Robust machine learning segmentation for large‐scale analysis of heterogeneous clinical brain MRI datasets. Proc. Natl. Acad. Sci. U.S.A. 2023;120 (9) e2216399120, https://doi.org/10.1073/pnas.2216399120. Figure S3. Relaxometr [file NBM-38-e70056-s001.docx]

# Supporting information

## S1: Simulated FID data


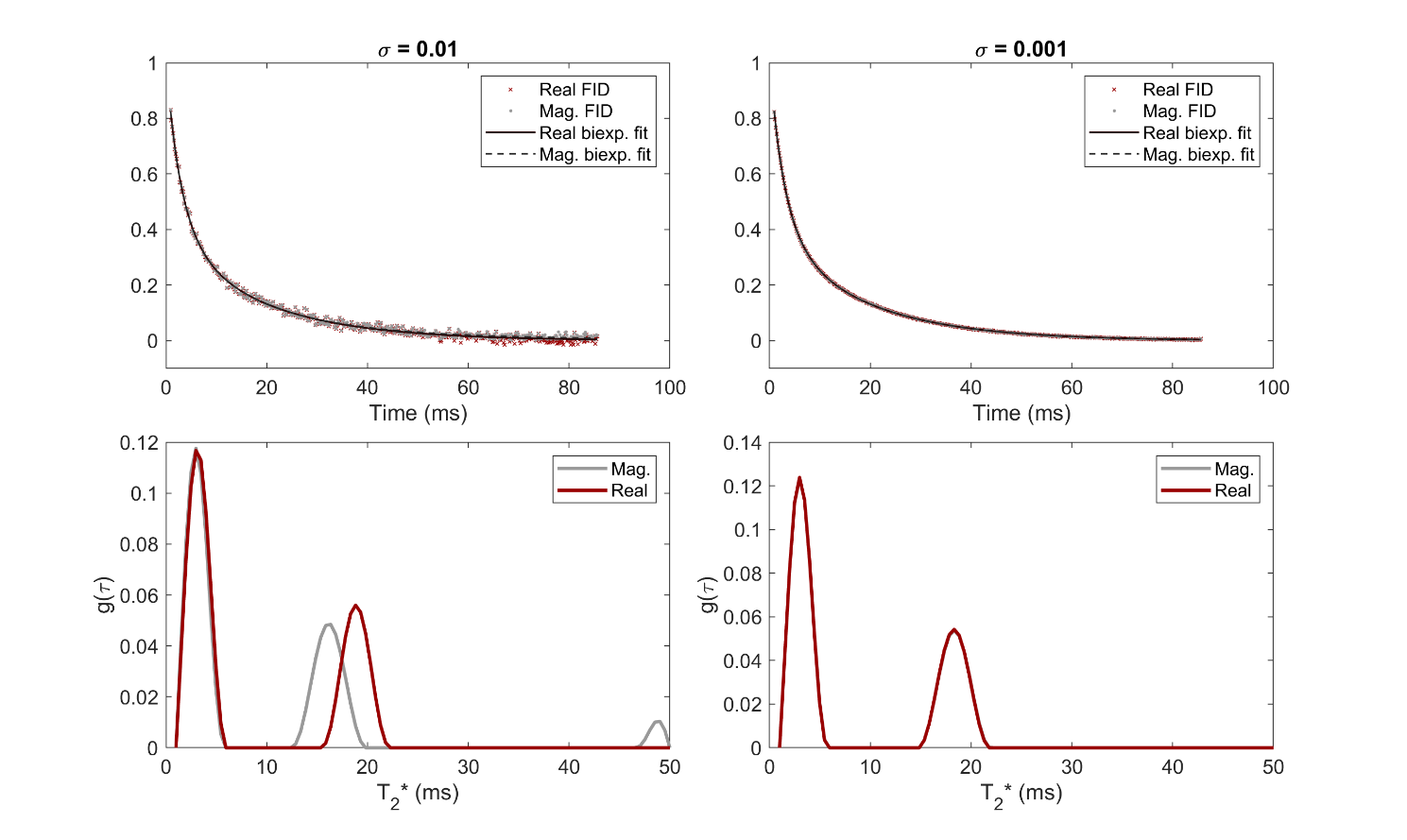


**Figure S1** To verify accuracy of the $T_{2}^{*}$ fitting method, a biexponentially decaying signal was simulated with: $T_{2s}^{*}$ = 3 ms, $T_{2l}^{*}$ = 18 ms, $f$ = 0.6 ms and additive complex Gaussian noise of zero mean and standard deviation $\sigma$ = [0.001, 0.01], simulating both “low SNR” and “high SNR” regimes. Sequence parameters (TE, bandwidth, no. samples) were chosen in accordance with the acquisition protocol. Fitting was performed on the real component and the magnitude of the signal. Above, the first row of plots show the simulated signals and fitted biexponential decays; the second row of plots show the fitted $T_{2}^{*}$ spectra; columns show different SNR levels. The importance of utilising the real signal component is illustrated, as magnitude data induces a bias in $T_{2l}^{*}$ estimates at lower SNR (and therefore sodium concentrations).

**Table S1** This table summarises the numerical results of the simulation experiment described in Figure S1. Fitted parameters are in good agreement with theoretical ones ($T_{2s}^{*}$ = 3 ms, $T_{2l}^{*}$ = 18 ms, $f$ = 0.6 ms), apart from the case of fitting on magnitude data at low SNR. Results imply the fitting method is of good accuracy.

| ***Sample S1:* σ *= 0.01*** | |  |  |  |  |
| --- | --- | --- | --- | --- | --- |
|  | $T_{2s}^{*}$ (ms) | ± | $T_{2l}^{*}$ (ms) | ± | $f$ |
| Real | 3.0 | 1.4 | 16.3 | 1.6 | 0.63 |
| Magnitude | 3.0 | 1.3 | 18.8 | 1.8 | 0.61 |
|  |  |  |  |  |  |
| ***Sample S1:* σ *= 0.001*** | |  |  |  |  |
|  | $T_{2s}^{*}$ (ms) | ± | $T_{2l}^{*}$ (ms) | ± | $f$ |
| Real | 3.0 | 1.3 | 18.3 | 1.8 | 0.62 |
| Magnitude | 3.0 | 1.3 | 18.3 | 1.8 | 0.62 |

## S2: In vivo ^23^Na MRI regions of interest (ROIs)


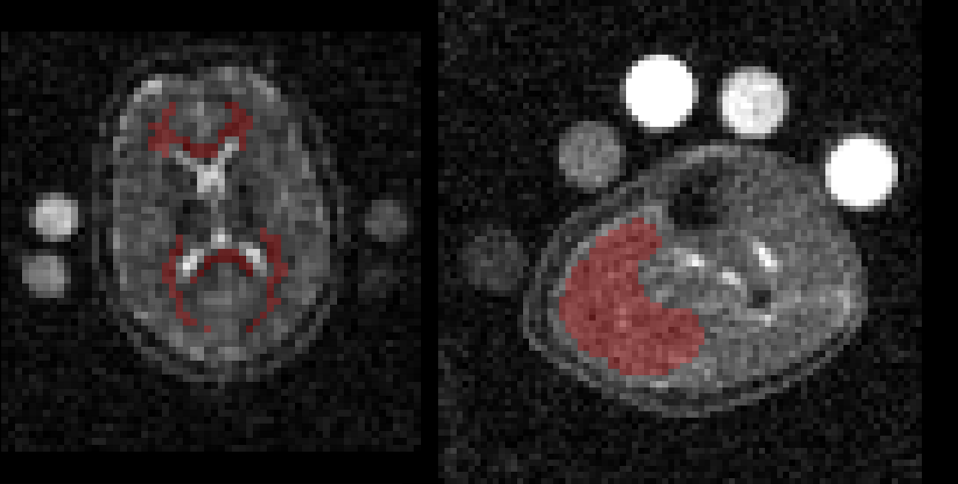


**Figure S2** Overlays of the analysis ROIs utilised for aTSC calculations on ^23^Na MRI images of the human brain (left) and human calf muscle (right). For the brain, a white matter specific ROI of 107 voxels was derived by applying an automatic segmentation algorithm (SynthSeg, <https://github.com/BBillot/SynthSeg>^[[1]](#footnote-1)^) to an additionally acquired T_2_ weighted ^1^H scan in the same image space. The resulting probabilistic segmentation was resampled and thresholded at 0.95 likelihood for white matter. For the leg, a conservative ROI of 366 voxels was manually drawn inside muscle tissue.

## S3: Relaxometry data of all samples


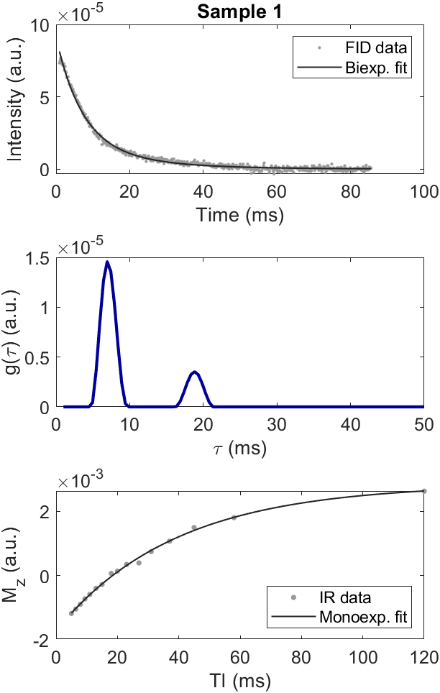

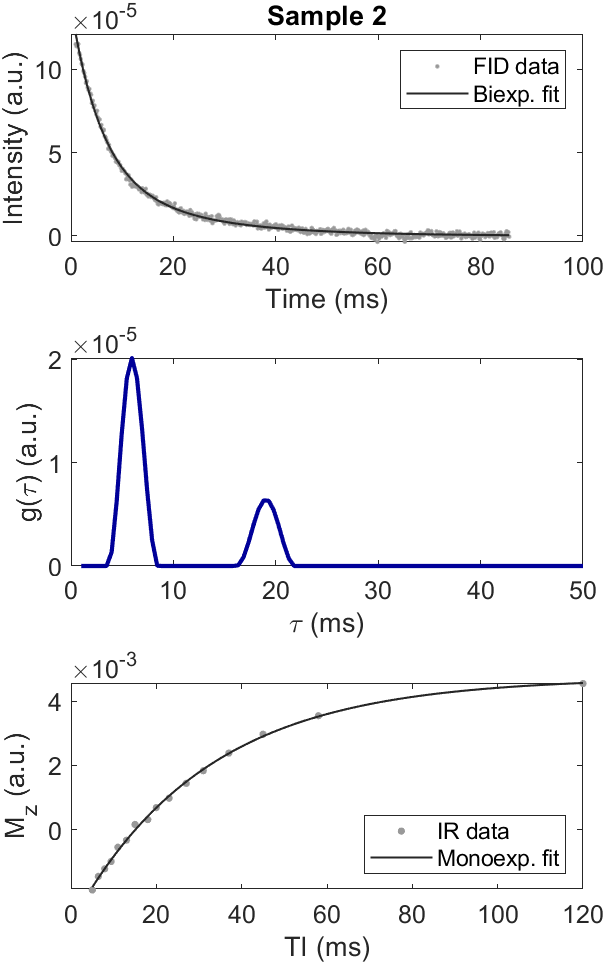

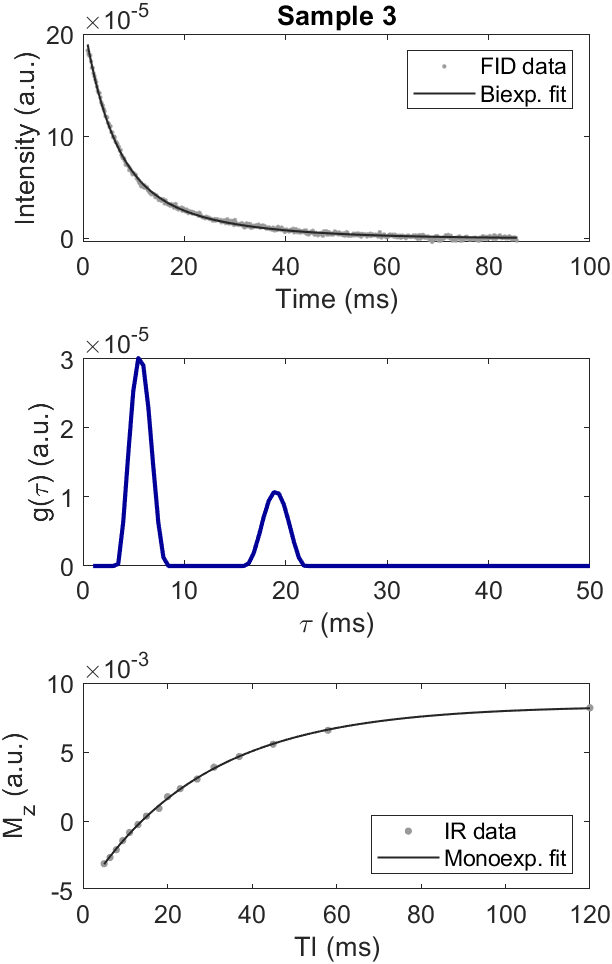

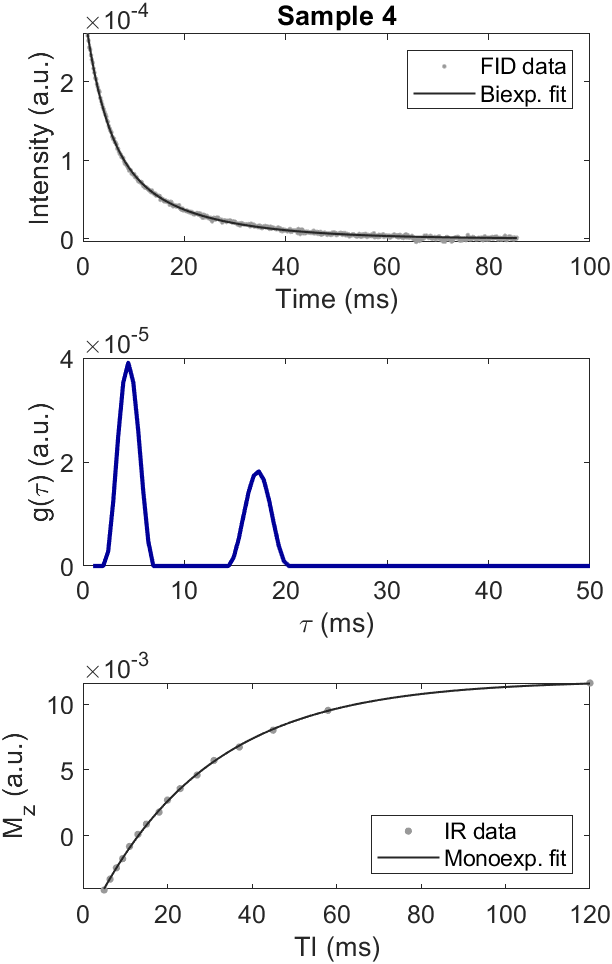

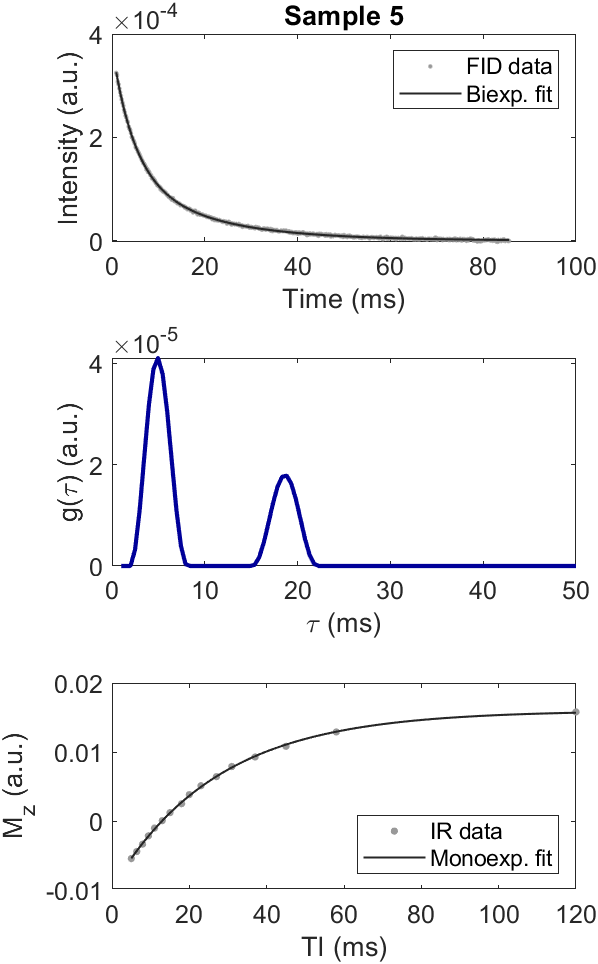

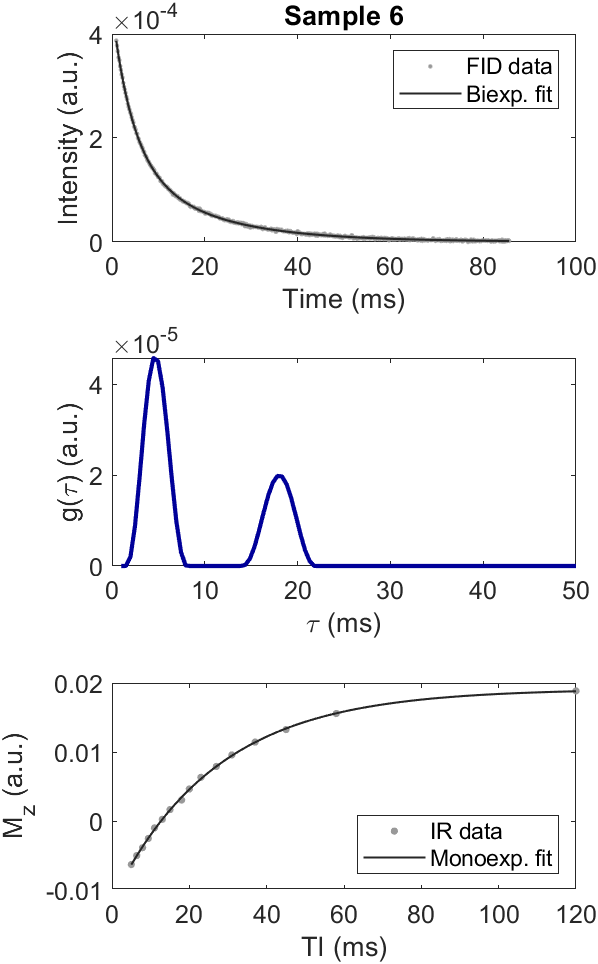

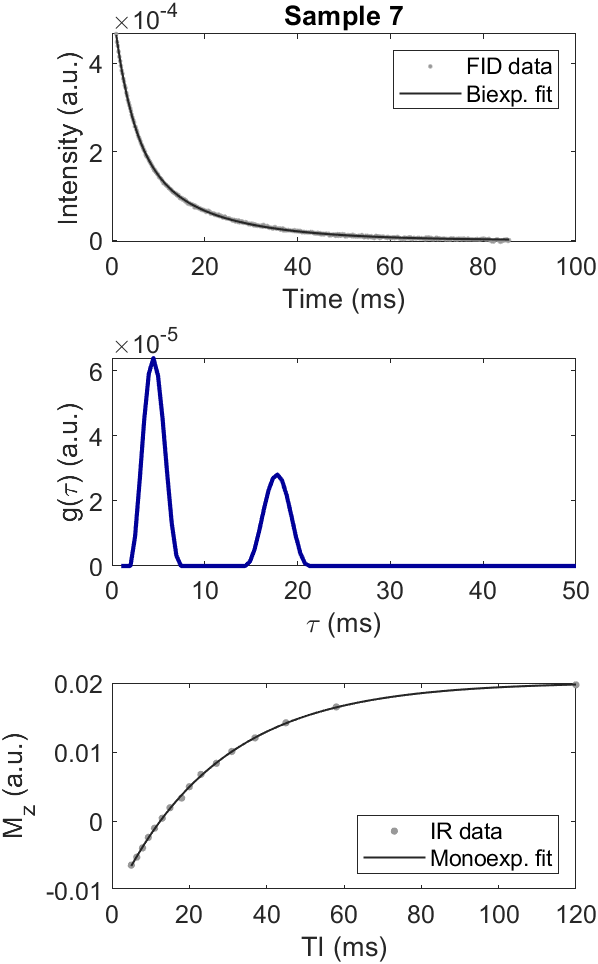


**Figure S3** Relaxometry data and fitted curves for all samples 1–7. Each tile of three plots shows the FID signal and biexponential fit (top row), the fitted spectrum of $T_{2}^{*}$ values (middle row) and monoexponential inversion recovery data and fit (bottom row).

## S4: Visual representation of relaxometry data


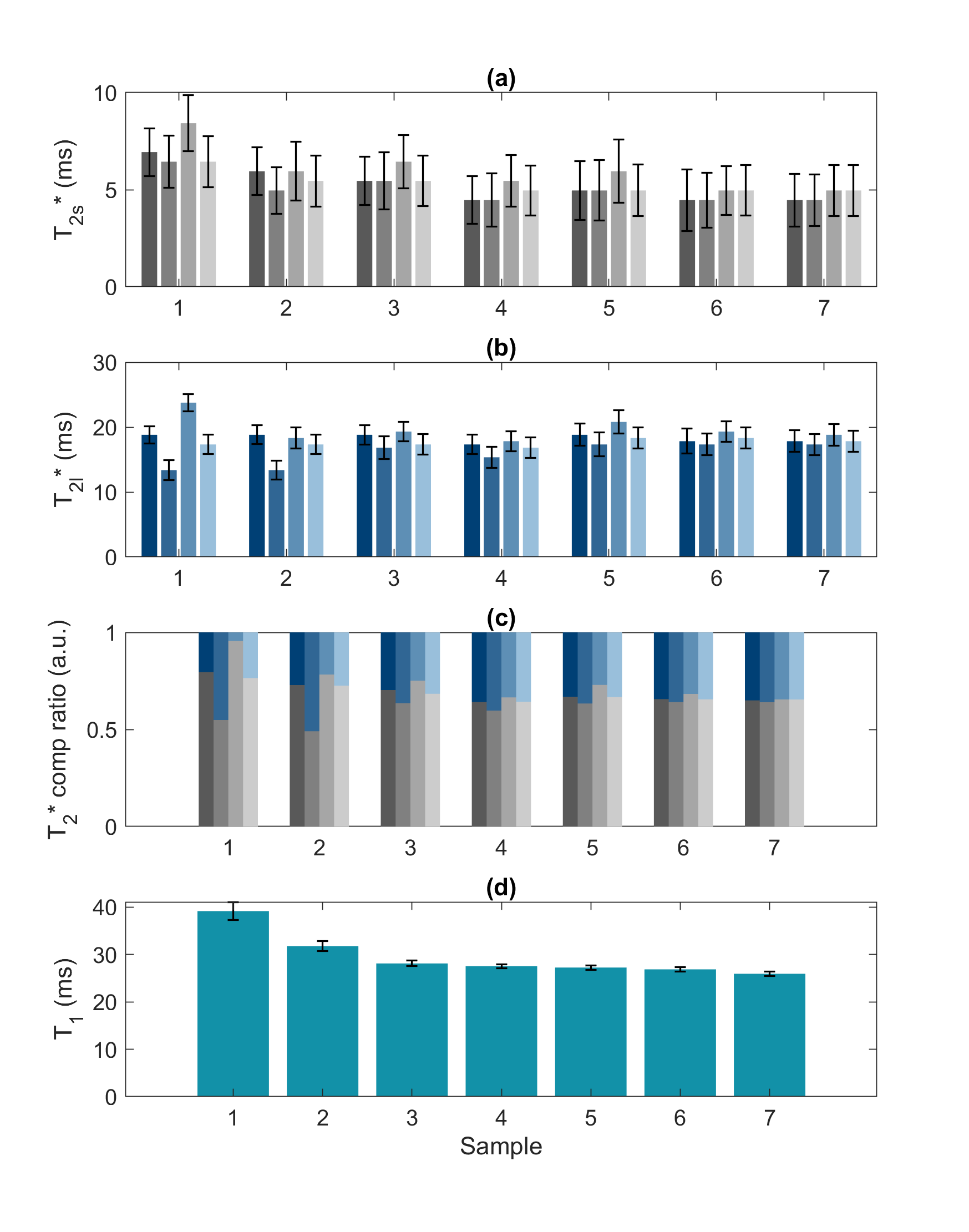


**Figure S4** A visual representation of the biexponential $T_{2}^{*}$ and monoexponential $T_{1}$ relaxometry results for all phantom samples 1-7, as listed in Table 3. Charts (a)–(c) show fitted $T_{2s}^{*}$, $T_{2l}^{*}$ in grey and blue, as well as component ratios, for 4 longitudinal repeats at 0, 6, 12 and 14 months. Error bars indicate the widths of fitted spectrum peaks. Chart (d) shows fitted $T_{1}$. Error bars indicate standard errors of the fit. Bars represent individual measurements.

## S5: Samples at different PAG concentrations

**Table S5** For initial, exploratory experiments (otherwise not reported), PAG samples were prepared at different concentrations of gelling agents. This table reports the results for $T_{2}^{*}$ relaxometry of two such samples: X1, prepared with 3% gelling agent, of which 5% was cross-linker; X2, prepared with 10% gelling agent, of which 5% was cross-linker. Both were prepared at 85mM nominal sodium concentration. Fitted biexponential $T_{2}^{*}$ values differ significantly from those found in the prototype samples analysed as part of this study, demonstrating that PAG could be prepared at different concentrations to generate phantoms with unique relaxation properties. The origin of such variations of $T_{2}^{*}$ with PAG concentration should be a subject of further investigation.

| ***Sample X1: 85mM, 3% gelling agent, of which 5% cross-linker*** | | | | | |
| --- | --- | --- | --- | --- | --- |
| Repeat | $T_{2s}^{*}$ (ms) | ± | $T_{2l}^{*}$ (ms) | ± | $f$ |
| 1 (month 0) | 5.5 | 1.7 | 23.8 | 3.7 | 0.07 |
|  |  |  |  |  |  |
| ***Sample X2: 85mM, 10% gelling agent, of which 5% cross-linker*** | | | | | |
| Repeat | $T_{2s}^{*}$ (ms) | ± | $T_{2l}^{*}$ (ms) | ± | $f$ |
| 1 (month 0) | 10.9 | 3.0 | 31.7 | 4.7 | 0.39 |

## S6: B_0_ inhomogeneity map of individual sample

**Figure S6** A masked B_0_ field map showing offsets to the sodium resonance frequency across the volume of a PAG phantom vial, following a 2^nd^ order shim routine as applied before non-selective spectroscopic measurements. Particularly the top of the vial, which protrudes from the sample holder, contains a greater spectrum of precession frequencies. The field map was acquired and reconstructed using a vendor provided B_0_ field mapping protocol (for ^1^H, 1 ms echo spacing, rescaled to the ^23^Na frequency).

1. Billot B, Magdamo C, Cheng Y et al. Robust machine learning segmentation for large-scale analysis of heterogeneous clinical brain MRI datasets. *Proc. Natl. Acad. Sci. U.S.A.* 2023;120 (9) e2216399120, https://doi.org/10.1073/pnas.2216399120. [↑](#footnote-ref-1)
